# The Modulation of Mimicry by Ethnic Group-Membership and Emotional Expressions

**DOI:** 10.1371/journal.pone.0161064

**Published:** 2016-08-24

**Authors:** Birgit Rauchbauer, Jasminka Majdandžić, Stefan Stieger, Claus Lamm

**Affiliations:** 1 Social, Cognitive and Affective Neuroscience Unit, Department of Basic Psychological Research and Research Methods, Faculty of Psychology, University of Vienna, Liebiggasse 5, 1010, Vienna, Austria; 2 Cognitive Science Research Platform, University of Vienna, Universitätsstraße 7, 1010, Vienna, Austria; 3 Institute of Normal and Pathological Physiology, Slovak Academy of Sciences, Bratislava, Slovakia; 4 Research methods, Assessment and iScience, Department of Psychology, University of Konstanz, Konstanz, Germany; 5 Department of Basic Psychological Research and Research Methods, Faculty of Psychology, University of Vienna, Liebiggasse 5, 1010, Vienna, Austria; University of Bologna, ITALY

## Abstract

Mimicry has been ascribed affiliative functions. In three experiments, we used a newly developed social-affective mimicry task (SAMT) to investigate mimicry´s modulation by emotional facial expressions (happy, angry) and ethnic group-membership (White in-group, Black out-group). Experiment 1 established the main consistent effect across experiments, which was enhanced mimicry to angry out-group faces compared to angry in-group faces. Hence the SAMT was useful for experimentally investigating the modulation of mimicry. Experiment 2 demonstrated that these effects were not confounded by general aspects of response conflict, as a Simon task resulted in different response patterns than the SAMT. Experiment 2 and pooled analysis of Experiments 1 and 2 also corroborated the finding of enhanced mimicry to angry out-group faces. Experiment 3 tested whether this effect was related to perceptions of threat, by framing angry persons as physically threatening, or not. Selective enhancement of mimicry to out-group persons framed as physically threatening confirmed this hypothesis. Further support for the role of threat was derived from implicit measures showing, in all experiments, that black persons were more strongly associated with threat. Furthermore, enhanced mimicry was consistently related to response facilitation in the execution of congruent movements. This suggests that mimicry acted as a social congruency signal. Our findings suggest that mimicry may serve as an appeasement signal in response to negative affiliative intent. This extends previous models of mimicry, which have predominantly focused on its role in reciprocating affiliation. It suggests that mimicry might not only be used to maintain and establish affiliative bonds, but also to ameliorate a negative social situation.

## Introduction

Picture yourself in a café, observing interactions taking place: you might spot some people who, over their coffee and engaged in conversation, interactively align their postures and gestures. This tendency to mimic each other’s behavior (i.e. behavioral mimicry) has been referred to as the “Chameleon-effect” [1]. Notably, behavioral mimicry occurs automatically, with neither the deliberate intent to imitate nor the conscious awareness of being imitated [1, 2, 3]. This automaticity presumably results from (automatic) motor resonance processes, which are based on the direct link between motor representations of actions and the perceptual representations of their execution [4, 5, 6]. Importantly, behavioral mimicry seems to have positive effects on social interactions, as it increases social cohesion and prosocial behavior. Hence it has been suggested that behavioral mimicry constitutes an implicit affiliative signal [3, 7, 8, 9].

In line with this notion, it has been shown that people do not mimic others invariantly; rather, the extent to which they do so varies according to their affiliative motivations [1, 2, 3, 7, 9–15]. For example, behavioral mimicry was enhanced when a goal for affiliation was actively primed [7], when inclusion into a social group had to be regained [11], or if the prior affiliation between the interacting individuals was high [1, 2, 3, 7, 11]. Conversely, behavioral mimicry has been found decreased when interaction partners are stigmatized [12] or disliked [13]. Findings such as these suggest that behavioral mimicry can be modulated by a variety of social and motivational factors. Moreover, mimicry has been shown to also have direct positive effects on reduction of prejudices [16] and increase in empathy [17] in dealing with out-groups.

### Automatic Imitation as a Laboratory Model of Mimicry

It has been suggested to use automatic imitation tasks, which rely on stimulus-response compatibility (SRC) effects, as laboratory models of behavioral mimicry [6]. Similar to the Chameleon effect investigated using more naturalistic experimental approaches (for example see [1, 8, 9, 11, 13, 14], automatic imitation has also been found to be malleable by socio-affective factors such as social status of the mimickee [15], pro- and antisocial priming [18], eye-contact [19], and valence of emotional primes [20]. One of the main advantages of automatic imitation paradigms, compared to the naturalistic experimental approach by which the Chameleon effect has been studied, is that they allow investigating modulations of mimicry by social factors with higher experimental control, using for instance within-subject designs or more quantifiable and sensitive outcome measures, such as responses times. Moreover, they also allow elucidation of the neural mechanisms engaged in mimicry´s malleability [15, 21–25]. Automatic imitation tasks, such as the imitation-inhibition task developed by Brass and colleagues [5], exploit the fact that observation of another person’s (task-irrelevant) movement affects motor execution of a required response, by generating an automatic tendency to imitate the other person’s movement in the observer [5, 6]. Since we used automatic imitation as a laboratory model of mimicry, we will also henceforth refer to automatic imitation investigated in such tasks as *mimicry*. Responses in these tasks are facilitated (as expressed by faster response times) when the observed movement is congruent with the required response (further referred to as the process of *response facilitation*). Perceiving an incongruent task-irrelevant movement slows down reaction time, as automatic motor resonance processes have to be blocked (further referred to as the process of *response inhibition*) [5]. In line with previous studies, using the mean difference in response time between incongruent and congruent trials as a measure of interference [26], mimicry regulation [22] or self-other distinction [27], we operationalized this difference measure as the *mimicry effect*. This mimicry effect thus accounted for both response inhibition and facilitation processes. Nevertheless, we also carried out separate analyses of response facilitation and inhibition to account for the processes separately.

Major advantages of automatic imitation paradigms as laboratory models of mimicry, are that first, they allow the establishment and extension of models of mimicry and its modulation by social context. Second, they allow disentangling the involvement of the processes of response facilitation and response interference in the overt mimicry effect. This allows also for direct comparison of movement congruency effects (congruent trials) against a control condition of movement incongruency, and a non-movement baseline condition, which naturalistic mimicry set-ups are usually lacking (e.g. [1, 3, 8, 9]).

### The Social-Affective Mimicry Task (SAMT)

We set-up a modified version of the imitation-inhibition task allowing us to investigate the effects of social-affective factors on mimicry. Our aim was to propose an extension of social cognition models of mimicry accounting for its functional malleability by distinct affiliative goals. More specifically, we propose that mimicry might not only be used to signal and reciprocate affiliation in response to positive affiliative signals. In the present article, we suggest that mimicry might also represent an affiliative signal for appeasement in response to counter-affiliative signals, signaling a potential threat. We combined the imitation-inhibition task [5], with simultaneously presented face stimuli which were varied with respect to ethnic group membership (White vs. Black) and emotion expressions (happy vs. angry) [28]. (Note, that we prefer to use the term "ethnicity" instead of "racial", as a more neutral description of socio-cultural and physical, but not biological-genetic, differences between individuals. The term "racial" and its use may have some problematic connotations in public use (e.g. measures against racial groups based on presumed "biologically determined" inferiority) [29].

### Study Aims

Our study had three main aims. First, we aimed to demonstrate mimicry´s malleability by social-affective context, i.e. emotions expressed by in- or out-group members, via its proposed laboratory “substitute” [6] of automatic imitation. As previous evidence had shown modulation of mimicry by social cues, our second and more eminent goal was to assess the tailored regulation of mimicry for the achievement of distinct affiliative goals. Our main hypotheses were that mimicry can be flexibly regulated to either reciprocate positive affiliative signals, such as when responding to a smiling interaction partner—or to support appeasement in response to counter-affiliative signals, such as the ones conveyed by an angry and potentially threatening interaction partner. Our third aim was to investigate whether mimicry´s tailored regulation is predominantly driven by processes related to motor response facilitation.

Overall, our study’s main objective was to propose an extended view of the affiliative functions of mimicry. This view is that mimicry serves as an implicit affiliative signal, which adaptively regulates behavior goal-directedly, to not only maintain or intensify existing social cohesion, but also to ameliorate an unfavorable social situation.

### Regulation of Mimicry by Distinct Affiliative Goals—Reciprocation of Affiliation and Appeasement?

Establishing and maintaining stable and cohesive social groups has high impact on human life: Individuals benefit from social support in many ways, including amelioration of mental and physical health [30, 31, 32]. Thus social group membership is likely to affect affiliative intent in the mimicker. Recognizing a member of the same social group, be it the same ethnical group [10, 33, 34] or someone supporting the same soccer-club [35], seems to elicit a motivation to affirm this shared identity via affiliative displays [34], and aiming amongst other things to secure mutual cooperation [10, 34, 36]. Additionally, it could serve to communicate group boundaries to groups stereotyped as threatening, as has been shown for Black people [34, 36, 37, 38, 39]. Considering this, and in line with the notion that mimicry signals affiliative intent, it can be suggested that, in the absence of other context information, such as the emotional expression of the interaction partner, mimicry may be higher in response to in-, as compared to out-group members [40].

Yet, besides social group membership, affiliative intent can also be discerned from emotion expressions: expressions of happiness, such as in a smile, can be assumed to inherently signal affiliative intent, whereas an angry facial expression implies aggression and conveys counter-affiliative intent [10, 34, 41, 42, 43]. Importantly, Bourgeois & Hess (2008) [10] have shown that happy facial expressions were mimicked (facial mimicry) equally for both in- and out-group members, as reciprocating affiliation comes at low cost. Therefore, if affiliative intent is reciprocated via mimicry, observing individuals with happy facial expressions, regardless of group membership, should also evoke more behavioral mimicry, as measured via the SAMT, than angry facial expressions. Thus, so far, it remains open, whether the smile’s affiliative signal would outweigh group differences and lead to a reciprocation of the affiliative signal via behavioral mimicry.

So far, the modulation of mimicry by affiliative behavior has mostly been studied in the context of securing social cohesion within existing groups or establishing new social bonds [1, 8, 9]. However, recent evidence shows that mimicry was increased after experiencing social rejection [11] and to non-social stimuli carrying negative valence [20]. It is has been shown that implicit perception of the social status of the out-group drives the modulation of instructed imitation of gestures of out-group members [44, 45]. Thus, it seems that implicit perceptions, as well as the relevance of social signals for the own current social and affiliative goals guide mimicry. Despite mimicry potentially being regulated flexibly for reciprocation of affiliation or appeasement, in the SAMT the magnitude of the mimicry effect appears as a uniform measure (i.e. number). Yet, it has been shown that distinct neural processes guide this flexible regulation of mimicry towards distinct affiliative goals [22]. Moreover, in intergroup contexts it has also been shown that being mimicked enhances empathy towards out-group members [17]. Also, explicitly mimicking Black out-group members reduces implicit racial prejudices [16]; an effect that has also been found when inducing illusion of ownership of a Black hand through a Rubber Hand Illusion in White participants [46]. Thus, it has been shown, that mimicry may have positive influences on intergroup relations. In the present article it is suggested that mimicry, in its affiliative function, may also be used to express appeasement and conciliation towards a potentially threatening other [47, 48, 49]. As such, the behavioral goal might be to soothe potentially threatening interaction partners—in particular if the opponent´s potential harm-inflicting abilities are judged higher than one´s own [50]. This might be the case for e.g. Black people, as it has repeatedly been observed that White, participants implicitly stereotype Black out-group members as more threatening [34, 36, 37, 38, 39, 50], including to one’s “physical safety” [50]. Appeasement behavior has also been documented in other primates. For instance, primates may in some specific settings show affiliative behaviors such as embracing to prevent aggression and to de-escalate conflict in social interactions, rather than withdrawal or fighting behavior [47, 49]. In line with these observations, mimicry might aim to signal appeasement and, in turn, evoke more empathy [17] in the opponent, thus leading to de-escalation and the soothing of a conflict. Therefore, we propose that the counter-affiliative signals from potentially threatening others (in our case angry out-group faces) might result in increased mimicry, in line with its supposed implicit appeasement function.

### Specific Regulation of Response Facilitation Driving the Modulation of the Mimicry Effect?

As outlined above, the third aim of our study was to investigate whether the regulation of mimicry in accordance to distinct affiliative goals was specifically related to enhanced facilitation of the motor response in congruent trials, rather than to stronger interference effects in incongruent trials. This hypothesis is based on the fact that the Chameleon effect reflects engaging in the same (congruent) action or behavior as one’s interaction partner. On a process-level, response facilitation on congruent trials in mimicry tasks could serve a social-feedback signaling function, achieved by the enhanced display of congruency with the other and thus represent a social congruency signal. Response inhibition, in contrast, as the blocking of a resonant motor response, might potentially lack the social communicative value of feeding back affiliative signals to the interaction partner. Indeed, previous evidence of a modulated facilitatory effect on congruent trials, but less so on incongruent trials, comes from a study combining another mimicry paradigm with the manipulation of either direct or averted gaze [19].

### Current Research and Theoretical Contributions

We performed a series of three behavioral experiments, which both included internal replications of the SAMT, as well as additional tasks targeted at ruling out potential alternative explanations for the found effects. Experiment 1 was used to establish the paradigm and the proposed effects of social-affective cues on mimicry and response facilitation. In Experiment 2 we investigated whether the observed mimicry effects were specifically related to motor resonance, by comparing mimicry effects with effects related to response conflict induced by spatial stimulus-response (in)congruency (Note that for consistency with the mimicry task we prefer, and will further use; the term (in)congruency over (in)compatibility). Due to the structural equality of the SAMT in Experiment 1 and 2 we pooled the SAMT data of Experiment 1 and 2 (controlling for effects of the experiment). This allowed to enhance our statistical power by minimizing the confidence intervals (see Cumming, 2013 [51] for arguments for pooling data of even 2 studies for enhanced analytical power; as well as [52] for best practice recommendations in social psychology). Experiment 3 aimed to specifically investigate whether manipulating the perceived threat of physical safety posed by angry out-group members would elicit enhanced mimicry of arbitrary finger movements, and thus support the notion that mimicry effects in this condition reflect attempted appeasement.

Thus, the present work could yield conceptual contributions to social cognition models of mimicry in social interactions: First, by extending the view of mimicry from an affiliative signal serving to establish and maintain favorable social bonds, towards a thus far largely overlooked social function, which is to soothe and de-escalate a potential conflict by appeasement. Second, by investigating the regulation of mimicry specifically reflected in response facilitation, as a social feedback signal of congruency or response inhibition, as withholding of social feedback.

## Experiment 1

The main aim of Experiment 1 was to provide a first step in assessing the malleability of mimicry in response to differences in group-membership (in-group vs. out-group) and emotional expressions (happy vs. angry). As outlined above, we hypothesized a flexible regulation of mimicry for both reciprocation of affiliation, as well as appeasement. Our specific hypothesis regarding the reciprocation of affiliation was open, since a smile, as mutual group membership, usually signals affiliative intent, which might equally evoke the wish for reciprocation via mimicry. Our specific hypothesis regarding appeasement was that mimicry may be enhanced towards angry out- as compared to angry in-group members, as anger might signal aggression and Black out-group members have (implicitly) been associated with enhanced physical threat. Third, we predicted that modulation of the mimicry effect due to the affiliation-related social-affective manipulation in the task would be mainly driven by response facilitation. We expected that congruent trials would be modulated in response to the different conditions, whereas incongruent and baseline trials would be unaffected by the task-irrelevant presentation of faces.

### Methods

#### Participants

Sixty-two right-handed, White students took part in this study for course credit or financial compensation of 10 Euros (43 female, 19 male; *mean age*: 22.07 years, *SD* = 2.8). The experiment was approved by the ethics committee of the University of Vienna and performed in accordance with the ethical standards defined in the 1964 Declaration of Helsinki and its revision in 2013.

#### The social-affective mimicry task (SAMT)

**Stimuli and experimental design:** Participants first performed a modified version of the imitation-inhibition task [5]. In this task, participants have to lift their index and middle finger in response to number cues. Additionally, a hand is shown; upon appearance of the number cue this hand either performs a task-irrelevant finger lifting movement (either congruent or incongruent with the movement required by the cue), or remains still (baseline trials). Although the hand movements are irrelevant to the task, they have been shown to affect response times to the cue, speeding up responses to congruent cues, and slowing down responses to incongruent cues. We took the difference between response times to incongruent and congruent trials can be taken as a measure of the mimicry effect. A baseline condition measuring basic reaction times of the required response without interference of a concurrent movement (i.e. image of a still hand) allowed to investigate the influence of the observed (task-irrelevant) movement relative to the basic reaction time (i.e. faster reaction times for response facilitation and slower for response inhibition relative to baseline) [5]. Important for the present study, a baseline condition allows to directly investigate the influence of social context cues on basic reaction times, without a concurrent movement.

**Fig 1:**
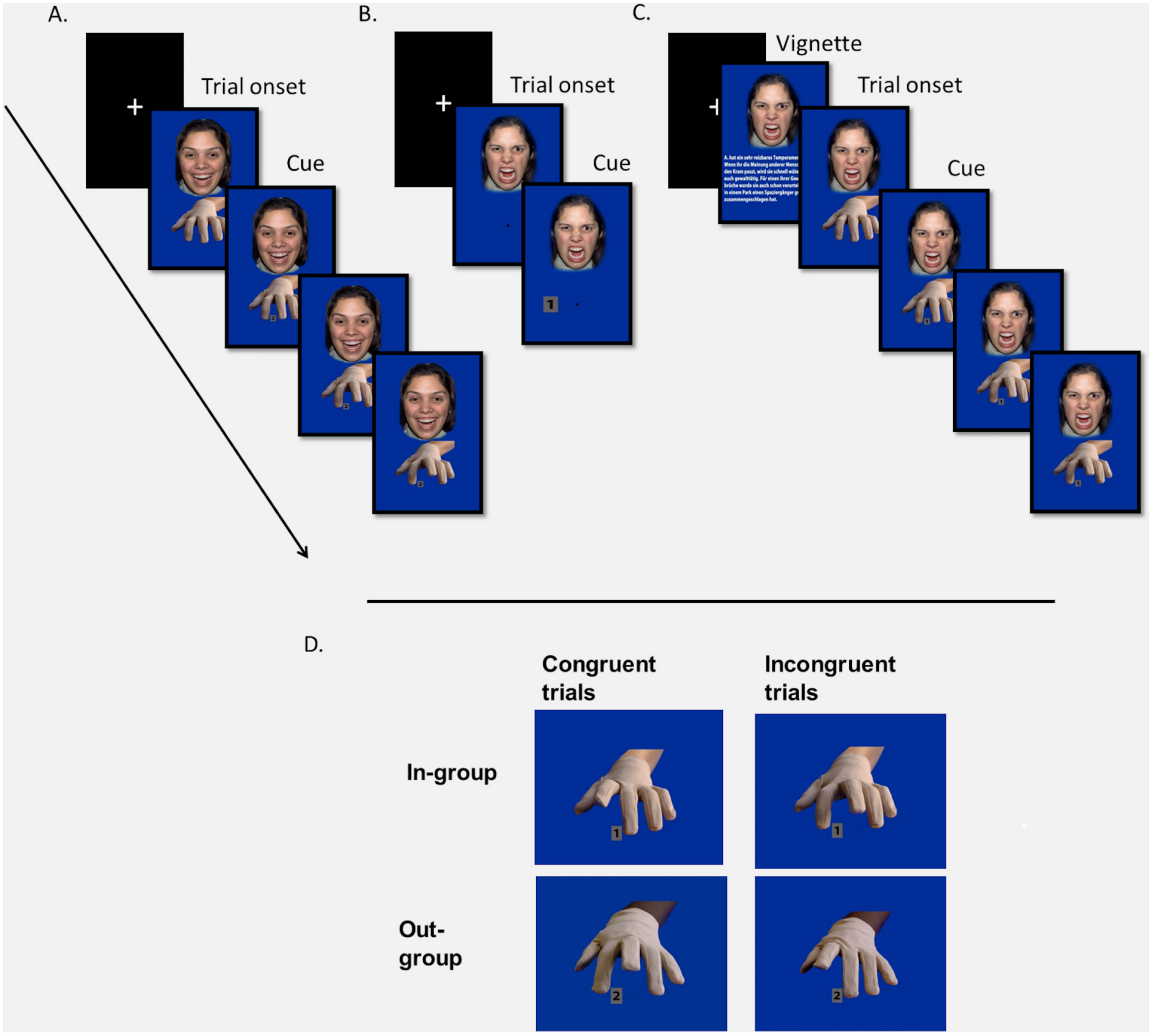
Experimental setup of different tasks and hand stimuli used in the present paper. A) Timeline of social-affective mimicry task (SAMT) (Face stimuli: NimStim set of facial expressions (Tottenham, 2009); Experiments 1 and 2; depicted are incongruent trials). B) Simon task (Experiment 2). C) SAMT with vignettes (Experiment 3, depicted are incongruent trials). D) In- and out-group hand stimuli in congruent and incongruent trials (Photos of hand shots taken by Birgit Rauchbauer, the permission for use of the hand shots was obtained by the hand models).

In the present study, task-irrelevant, female facial stimuli were shown above the hand stimulus (see Fig 1), depicting either in-group members (White faces) or out-group members (Black faces), with either a happy or an angry facial expression [28]. This resulted in a 2 x 2 x 3 factorial within-subjects design with factors Group (In-group, Out-group), Emotion (Happy, Angry) and Congruency (Congruent, Incongruent, Baseline).

Face stimuli were taken from the NimStim Set of Facial Expressions [28]. Happy and angry emotional expressions were shown by the same in- or out-group target, respectively (i.e., a total number of four female face stimuli from two targets were used). The hand stimulus was a frontal shot picture of a left hand (mirroring the participant’s right hand) wearing a beige cotton glove to avoid confounding effects on task performance and on mimicry due to perceptual and attention differences between conditions (such as the visual contrast between the black number cues on a grey square with the color of the fingers surrounding the cues, or differences in perceiving or attending to movements of dark vs. bright objects). Skin color and hence ethnicity was however visible at the wrist (which was far from the number cue, Fig 1) and matched to the skin color of the presented face; participants were wearing the same beige glove. The target cue, a black “1” or “2” on a grey square, was presented between index and middle finger of the displayed hand.

Each trial consisted of four consecutive frames (see Fig 1). The first frame displayed the hand in resting position and was presented for 2,000 milliseconds (ms). After 2,000 ms, the cue was displayed, simultaneously (for congruent and incongruent trials) with a finger lifting movement induced by two consecutive frames of 34 ms each. The last frame, depicting the hand with the fully lifted finger, was presented for 1,232 ms, resulting in a trial length of 3,300 ms. In baseline trials, the hand remained still throughout the trial. Intertrial-interval was fixed at 2,700 ms.

**Procedure:** Stimuli were displayed on a PC running Presentation Version 0.61. Display resolution was 1,280 x 1,024 pixels (300 dpi). After reading and signing an informed consent form, including detailed instructions, participants started with practice trials consisting of 8 trials before proceeding with the actual task. Participants were seated at a free-viewing distance of approximately 50 cm and kept the keyboard number pad key “1” pressed with the index finger and the “2” key with the middle finger. They were instructed to lift the index finger in response to a “1” cue, and the middle finger in response to a “2” cue. Furthermore, instructions stated that all other stimuli presented were irrelevant to the task, thus the modulation the mimicry response by social-affective stimuli happened *implicitly*. We define implicitly as Greenwald et al. (1998): “(…) actions or judgements that are under the control of automatically activated evaluation, without the performer’s awareness of that causation.” (page 1464, [53]). We had a 2 (In, Out-group) x 2 (Happy, Angry) x 3 (Congruent, Incongruent, Baseline) x 2 (Index, Middle finger) task set-up with 10 trials in each of these conditions; condition order was randomly varied. This resulted in a total of 240 trials and a total duration of approximately 24 minutes.

**Data analysis:** Only data from correct trials were included in the data analysis. To account for outliers a winsorization procedure was applied to the subjects’ individual mean response time (RT) per condition and target cue before further statistical analyses were conducted. Mean RTs higher than the 75^th^ percentile plus 1.5 times the interquartile range of the conditions per target cue, respectively, mean RTs lower than the 25^th^ percentile minus 1.5 times the interquartile range of the conditions per target cue were replaced with the maximum, respectively the minimum mean reaction time (in ms) in the particular condition. For each of the four conditions, the size of the mimicry effect was calculated by subtracting participants´ mean RT on congruent trials from the mean RT on incongruent trials.

#### Attitudes towards blacks scale

To measure explicit ethnic bias, a German translation of the Attitudes Towards Blacks Scale [54] was administered after the social-affective mimicry task. It consisted of 20 statements on attitudes towards Black people that had to be rated on a seven-point scale ranging from “1” (strongly disagree) to “7” (strongly agree). A low mean score on the scale indicates negative explicit attitudes, and a high score denotes favorable explicit attitudes towards Black people.

#### Threat/security implicit association test (threat IAT)

In order to further test our interpretation of the SAMT findings, we used an adapted version of the Implicit Association Test (IAT; [53]), but, due to technical problems, only in a large subset (85.9%) of the sample (55 participants, 38 female, 17 male; *mean age* = 21.96 years; SD = 2.84). The IAT is a frequently used measure to investigate implicit ethnic bias (for a meta-analytic review, see [55]). We developed a threat-IAT to assess the strength of participants’ implicit associations between the target concepts in-group (Whites) and out-group (Blacks) and the attributes “Security” and “Threat”, respectively. The categories Security and Threat each consisted of five German nouns describing the concepts of Threat and Security matched for word length and valence (for Threat: fear, threat, violence, attack, danger; for Security: peace, protection, calmness, shelter, security).

In line with the common approach (IAT [53]) the IAT consisted of serial presentation of five discrimination tasks. Participants were instructed to put their middle or index fingers on the “E”, respectively the middle finger on the “I” on the keyboard. They were asked to answer as fast as possible, and to disregard when they committed errors, in which case a red cross would appear. The first task was to sort a target picture of a White, respectively Black person into the categories of White, on the left hand side, and Black, on the right hand side. In the second part the target words describing Threat and Security had to be sorted into the categories of Security (left side), and Threat (right side). In the third task the target categories consisted of White and Security combined on the left side, and Black and Threat combined on the right side; both pictures of Blacks and Whites, as well as the target words describing Threat and Security had to be sorted into the correct category. In the fourth part the mapping of the target concept of group (i.e., Black and White) was switched with regard to the first task. This interchanged assignment of group was combined to the attributes (i.e., Threat and Security) in the fifth part resulting in the combined categories Black and Security on the left side and White and Threat on the right hand side. In line with past research, the IAT score was calculated based on the recommended *D*-measure [56]. High *D*-values represent a strong association of Blacks with Threat and White people with Security and low *D*-values represent a low association of Blacks with Threat and White people with Security.

Reaction times on the threat-IAT were winsorized to correct for outliers. Participants with more than 30% errors in one of the blocks were excluded from the analysis. In line with previous approaches, the *D*-measure [56] was calculated by first obtaining the latency standard deviation (*SD*) of reaction times of the combined fifth and the third task and subsequently dividing the reaction time (ms) difference (of fifth and third task) by this *SD*.

### Results

The winsorising procedure identified 18 datapoints (7.5%) of the subjects’ individual mean response time (RT) per condition and target cue as outliers, with mean RTs higher than the 75^th^ percentile plus 1.5 times the interquartile range of the conditions per target cue. These datapoints were thus replaced with the maximum mean reaction time (in ms) in the specific condition.

#### Social-affective mimicry task (SAMT)

**Fig 2:**
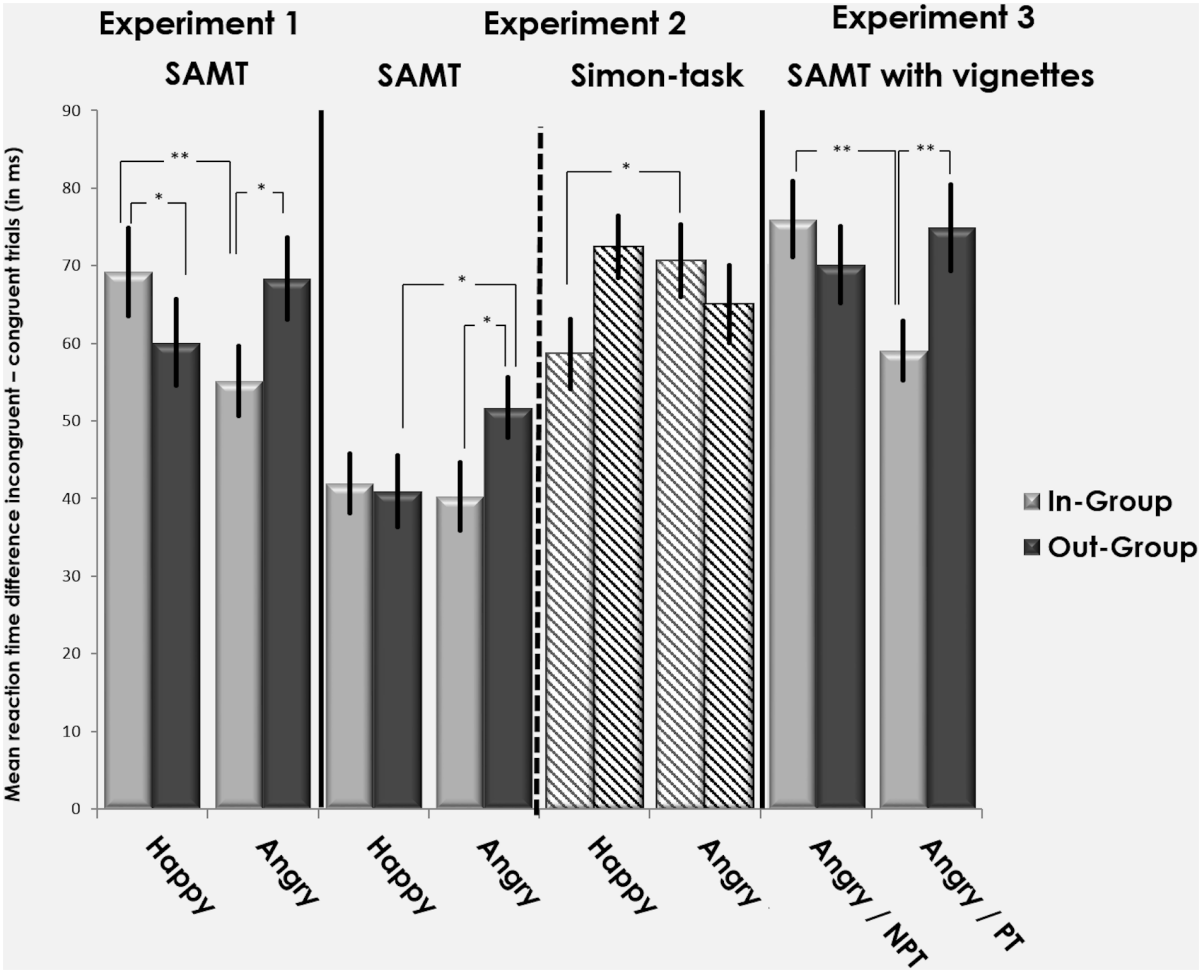
Social-affective mimicry task (SAMT, Experiment 1 and 2), Simon-task (Experiment 2) and SAMT with vignettes (Experiment 3). Bars represent the mean reaction time (RT) differences on incongruent and congruent trials of the social-affective mimcry task (Experiment 1 and 2) and Simon Task (Experiment 2); RT units are in ms; error bars represent standard error of the mean *(SE)*; * *p* ≤ .05, ** *p* ≤ .01, NPT = No Personal Threat, PT = Personal Threat.

**Mimicry effect:** Our main focus was to specifically test and substantiate our hypotheses about the modulation of the mimicry effect by the social-affective conditions. This was addressed with a two-way repeated measurement ANOVA with factors Group (In-group, Out-group) and Emotion (Happy, Angry) on the mean difference scores (i.e., RT on incongruent minus congruent trials). This revealed a significant Group x Emotion interaction (*F*(1,61) = 7.74, *p* = .007, partial η^2^ = .11) (see Fig 2), in the absence of significant main effects (all *p*s ≤ .365). In addition, we carried out planned pairwise comparisons (Bonferroni corrected significance level for the four comparisons p ≤ .0125). This revealed a trend significant higher mimicry effect in response to happy in-group than out-group facial expressions (*t*(61) = 2.11, *p* = .039; see Table 1 for details). The mimicry effect, by trend, was significantly higher in response to angry facial expressions shown by the out-group in comparison to the in-group member (*t*(61) = 2.46, *p* = .017; see Table 1 for details). Within the in-group condition, a higher mimicry effect was found in response to presentation of happy compared to angry facial expressions (*t* (61) = 2.84, *p* = .006), but no significant difference between these two conditions was found for the out-group stimuli (*p* ≥ .158).

**Table 1:** Results mimicry effect (difference measure mean RT incongruent minus congruent trials) and response facilitation (mean RT on congruent trials) with corresponding confidence intervals in the SAMT across all three experiments and the pooled samples of Experiment 1 and 2, as well as the Simon-task in Experiment 2, in ms; italic numbers referring to standard error of the mean *(SE)*.

*Experiment*	*Mimicry Effect*	*Confidence Intervals*	*Response Facilitation*	*Confidence Intervals*
**1) SAMT**				
Happy In-group	69.92 *(5*.*65)*	[58.62; 81.22]	461.82 *(8*.*28)*	[445.27; 478.38]
Angry In-group	55.12 *(4*.*50)*	[46.11; 64.12]	469.77 *(8*.*59)*	[452.60; 486.94]
Happy Out-group	60.11 *(5*.*50)*	[49.12; 64.12]	470.51 *(8*.*59)*	[453.49; 487,53]
Angry Out-group	68.33 *(5*.*26)*	[57.82; 78.85]	460.05 *(7*.*99)*	[444.07; 476.04]
**2) SAMT**				
Happy In-group	41.95 *(3*.*75)*	[34.44; 49.46]	449.16 *(6*.*24)*	[436.68; 461.64]
Angry In-group	40.27 *(4*.*36)*	[31.55; 49.00]	451.21 *(6*.*49)*	[438.23; 464.19]
Happy Out-group	40.95 *(4*.*57)*	[31.81; 50.10]	450.59 *(5*.*23)*	[440.13; 461.05]
Angry Out-group	51.73 *(3*.*90)*	[43.93; 59.52]	445.12 *(5*.*66)*	[433.80; 456.44]
**Combined sample: 1 and 2: SAMT**				
Happy In-group	55.93 *(3*.*04)*	[49.2; 62.67]	455.49 *(5*.*20)*	[445.20; 465.78]
Angry In-group	47.7 *(3*.*14)*	[41.49; 53.90]	460.50 *(5*.*39)*	[449.81; 471.17]
Happy Out-group	51.33 *(3*.*58)*	[44.24; 58.42]	460.55 *(5*.*01)*	[450.62; 470.47]
Angry Out-group	60.03 *(3*.*28)*	[53.54; 66.52]	452.59 *(4*.*91)*	[442.86; 462.31]
**Simon task**				
Happy In-group	60.85 (3.97)	[52.92; 68.78]	459.86 (6.35)	[447.17; 472.54]
Angry In-group	67.45 (3.49)	[60.48; 74.43]	451.85 (6.12)	[439.61; 464.10]
Happy Out-group	72.18 (3.92)	[64.22; 80.14]	456.07 (6.28)	[443.53; 468.62]
Angry Out-group	65.60 (5.01)	[55.58; 75.63]	460.62 (6.16)	[448.31; 472.93]
**3) SAMT with vignettes**				
Angry In-group / Personal Threat	59.06 *(3*.*84)*	[51.38; 66.74]	479.16 *(9*.*86)*	[459.40; 498.91]
Angry In-group / No Personal Threat	75.99 *(4*.*88)*	[66.21; 85.77]	470.65 *(8*.*45)*	[453.74; 487.56]
Angry Out-group / Personal Threat	74.91 *(5*.*54)*	[63.81; 86.01]	468.79 *(8*.*13)*	[452.52; 485,06]
Angry Out-group / No Personal Threat	70.11 *(4*.*90)*	[60.21; 80.00]	480.85 *(9*.*22)*	[462.38; 499.91]
Happy In-group	70.25 *(4*.*88)*	[60.47; 80.20]	480.05 *(9*.*77)*	[460.48; 499.61]
Happy Out-group	72.16 *(5*.*88)*	[60.39; 83.93]	477.97 *(8*.*81)*	[460.34; 495.61]

**Response facilitation:** The key aspect of this analysis was to test the hypothesis that the flexible regulation of the mimicry effect was based on response facilitation on congruent trials, rather than response inhibition in incongruent trials. First, mean RTs per condition were entered into a three-way repeated measurement ANOVA with factors Group (In-group, Out-group), Emotion (Happy, Angry) and Congruency (Congruent, Incongruent, and Baseline). This showed a significant main effect of Congruency (*F*(2,122) = 146.133, *p* < .001, partial η^2^ = .71). As expected, mean RTs on incongruent trials were higher than on congruent, (*t*(61) = 14.95, *p* < .001; incongruent: *M* = 529 ms, *SE* = 10 ms, congruent: *M* = 466 ms, *SE* = 8 ms) and baseline trials (*t*(61) = 7.51, *p* < .001; *M* = 500 ms, *SE* = 9 ms), thus replicating previous findings (for example [5]). We also obtained a significant Group x Emotion interaction (*F*(1,61) = 6.418, *p* = .014, partial η^2^ = .1), which is not in the focus of the present work. More importantly the results revealed a Group x Emotion x Congruency interaction (*F* (2,122) = 4.169, *p* = .018, partial η^2^ = .06).

Second, in order to specifically test whether the found Group x Emotion x Congruency interaction was driven by enhanced response facilitation on congruent trials, we conducted a two-way repeated measures ANOVA on congruent trials with factors Group (In-group, Out-group) and Emotion (Happy, Angry). This revealed a significant interaction Group x Emotion (*F* (1,61) = 16.47, *p* < .001, partial η^2^ = .21). Consecutive planned comparisons (Bonferroni corrected significance level p ≤ .0125) further showed a significant facilitation of the cued response (i.e., shorter RTs due to faster “facilitated” responses) when happy in-group compared to happy out-group facial expressions were shown (*t* (61) = -2.11, *p* = .01; see Table 1 for details). The facilitative effect was also significantly enhanced (i.e., faster responses) when angry facial expressions by the out-group member compared to angry in-group member expressions were presented (*t*(61) = -3.35, *p*, < .001; see Table 1 for details). Within in-group stimuli, a trend significant facilitative effect was found on presentation of happy compared to angry facial expressions (*t* (61) = -2.48, *p* = .016). For the out-group conditions a significant enhanced facilitation was found for angry faces compared to happy faces (*t* (61) = 3.36, *p* = .001). Further repeated measures ANOVAs on only the incongruent or the baseline trials with the factors Group x Emotion showed no significant differences (*p* > .146; see S1 File for details).

#### Threat/security implicit association test (threat-IAT)

The mean *D*-measure revealed a medium effect (*M* = 0.45, *SE* = 0.42; see Table 2 for details) of Black people being more associated with threat, whereas White people are more associated with security. Pearson correlations on the *D*-measures of individual subjects and their mimicry effect revealed no significant correlations (all *r*-*values* ≤ 0.11; all *p-values* ≥ 0.42). Also there were no further significant correlations between the individual *D*-measure of participants and the differences of specific comparisons between reaction times when angry out-group versus in-group stimuli were shown, neither with the corresponding comparison when happy stimuli were shown between groups (all *r*-*values* ≤ 0.04; all *p-values* ≥ 0.76).

**Table 2:** Scores on the Implicit Association Task across all three experiments.

*Experiment*	*CompatibleBlock (Block 3)*	*IncompatibleBlock (Block 5)*	*t*	*D-measure (SE)*
**1**	779.09 *(18*.*00)*	969.79 *(24*.*33)*	*t*(54) = 8.9**	0.45 *(0*.*04)*
**2**	739.89 *(11*.*18)*	878.73 *(24*.*60)*	*t*(59) = 6.36**	034 *(0*.*04)*
**3**	705.28 *(12*.*42)*	901.33 *(24*.*03)*	*t*(49) = 11.33**	0.51 *(0*.*04)*

Mean RT in ms in block 3 and 5; inferential statistic (T-value) and mean of D-measure; Italic numbers displaying standard error of the mean *(SE);*

** *p* ≤ .001

#### Attitudes towards blacks scale

The mean score on the Attitudes towards Blacks scale across all 62 participants revealed a neutral to favorable explicit attitude towards Blacks (*M* = 4.196, *SE* = 0.041). No significant correlation (Pearson correlation) of the scores on the Attitudes towards Black scale with the size of the mimicry, neither for white happy faces (*r* = 0.02; *p* = 0.9), nor any of the other conditions was found (all *r*-*values* ≤ -0.22; all *p-values* ≥ 0.09). Also there were no further significant correlations between the scores on the ATB of participants and the differences of specific comparisons between reaction times of displays of angry out-group versus in-group stimuli shown, neither with the corresponding comparison of presentation of happy stimuli between groups (all *r*-*values* ≤ 0.15; all *p-values* ≥ 0.25). Furthermore, there was no correlation (Pearson correlation) between scores on the Attitudes towards Black Scale and the individual *D*-measure (*r* = -0.07; *p* = 0.96).

### Discussion

Experiment 1 revealed significant interaction between the factors group and emotion for the mimicry effect. Further analysis revealed an increase in mimicry of arbitrary finger-lifting movements when participants were presented with stimuli of happy in-group (compared to happy out-group), as well as angry out-group members (compared to angry in-group). No difference was found for the presentation of happy as compared to angry out-group members (Fig 2). Moreover, results showed modulation of congruent trials, with higher response facilitation for happy in-group (as compared to happy out-group) members, and for angry out-group (as compared to happy out-group) members. Malleability of the mimicry effect was thus only driven by response facilitation, as shown by the significant modulation on congruent trials only.

The finding that happy in-group members evoke a more enhanced mimicry effect than angry in-, as well as happy out-group members, suggests that implicit mimicry is sensitive to the affiliative intention expressed by members of the in-group. A smile can communicate affiliation to the interaction partner [42], and in-group membership in itself may evoke a wish for affiliation [34, 35]. This finding therefore suggests that mimicry, might represent an implicit signal to behaviorally reciprocate affiliation, potentially enhanced through a wish for affiliation with happy in-group members. The absence of a difference in the magnitude of the mimicry effect between angry and happy in-group members, suggests that, as mentioned before, mutual group-membership may in itself elicit a wish for affiliation [34, 35, 40].

Nevertheless, results also showed that mimicry did not differ for happy out-group members, when compared to angry out-group members. This equally enhanced mimicry might suggest that also for happy out-group mimicry might be enhanced to reciprocate affiliation. This will be further investigated in experiment 2.

Presentation of angry faces from out-group members resulted in a higher mimicry effect than those of in-group members. Notably, this seems to fortify our hypothesis that mimicry, besides its function to support already smooth interactions, might be used as a signal to appease a threatening interaction partner as opposed to angry, but potentially not threatening, in-group member, for instance to de-escalate a potentially harmful conflict [49]. A similar argument comes from ethology observations of primate communication, stating that dependent on the context, affinitive signals may also be used with the goal to appease and prevent potentially harmful conflict [47, 48]. Since Black people have been implicitly associated with more threat [34, 36, 37, 38, 39, 50], and angry faces might signal aggression [42], faces of angry Black people might be experienced as a heightened threat, and thus particularly capture attention. The results of the Threat/Security Implicit Association Task (threat-IAT) are in line with this interpretation: we found a medium effect of implicit association of Black people with threat, and of White people with security. Explicitly participants exhibit neutral to favorable attitudes towards Blacks, indicating that the modulation of the mimicry effect and the response facilitation when angry out-group faces were presented were based on implicit perceptions.

The results revealed that the modulation of the mimicry effect was driven by the process of response facilitation in congruent trials. It is suggested that the process of response facilitation resembles a social signal of mutual congruency with the other. This congruency might enable feedback of affiliative signals in a social interaction.

With respect to our hypotheses, the results of Experiment 1, firstly, show a modulation of mimicry by social-affective cues. Second, the results reveal a tailored regulation of mimicry, which we propose to be related to reaching distinct affiliative goals. Notably, mimicry seems to not only be enhanced in situations of mutually reciprocated affiliation, but also in situations where a potentially threatening encounter calls for appeasement. Third, the adaptive regulation of mimicry for distinct affiliative goals was found to be based on response facilitation in congruent trials. Thus the results of Experiment 1 speak for a highly adaptive regulation of mimicry serving different social functions.

As mentioned before, increased mimicry by the display of an angry out-group face might also be due to enhanced attention towards this stimulus. It has also been suggested that threatening faces may lead to heightened efficiency in memory encoding [57]. Yet, importantly, the reaction times on incongruent and baseline trials were unaffected by the social-affective manipulation. If the observed effects would be purely driven by high saliency of and consequent heightened attention, and/or enhanced encoding efficiency towards the angry outgroup faces, this domain-general process would have caused RT effects not only in congruent, but also in incongruent and baseline trials. Nevertheless, we suggest that attention effects could play a role in facilitating mimicry on congruent trials by enabling fast signaling of motoric congruency.

However, the modulation of reaction times might also be explained by differences in stimulus congruency of the conditions, and thus result from domain-general priming processes of (in)congruency detection and conflict resolution rather than mimicry. More specifically, one might assume that in-group members showing positive emotions, and out-group members showing negative emotions, respectively, were perceived as more congruent, and that this has facilitated the response times. In order to rule out this alternative explanation, we conducted a second experiment (Experiment 2) in which participants in addition to the SAMT also performed a Simon task with the same social-affective face stimuli. Also we aimed to replicate the results of the SAMT in experiment 2, with a slight alteration in duration of stimuli presentation to investigate whether the results of experiment 1 could be replicated. This was done because, first, our hypothesis regarding enhanced mimicry in response to happy faces was originally open. Second, our hypothesis regarding enhanced mimicry towards angry out-group faces as compared to angry in-group faces was new and should be substantiated through replication. Experiment 2 also allowed to, in a later step, pool the SAMT data to enhance analytical power to fortify our conclusions.

## Experiment 2

In order to rule out alternative explanations for our RT effects, we aimed to test whether stimulus (in)congruency outside the motor domain would modulate response times in the same fashion as what we interpreted as mimicry-related effects in Experiment 1. That is, we wanted to rule out the alternative explanation that the RT findings of Experiment 1 reflected domain-general stimulus (in)congruency effects. We replicated the SAMT of Experiment 1, but added a variant of the Simon task combined with our socio-affective stimuli to assess domain-general stimulus-response (in)congruency effects of our manipulation. Furthermore, we aimed to test the stability of the effect in Experiment 1 for replication purposes, by altering the experimental set up and presenting the Simon Task first. Furthermore, we also aimed to test whether the found effect would replicate with shorter presentation of the task-irrelevant social-affective stimuli. For this we slightly reduced the presentation duration of frames 1 and 4 (as in [22]).

The Simon effect, which describes a faster response to congruent stimulus-response mappings [58] (see also [59]), represents a measure of cognitive response conflict caused by spatial (in)congruency between task-irrelevant stimuli and required responses [59]. A typical Simon task exploits the fact that the (task-irrelevant) spatial location of a cue directly affects reaction times, resulting in faster reaction times when the spatial locations of cue and response are congruent (i.e. congruent trials), and in slower response when they are incongruent. Thus, the Simon task served as a control task to assess whether the effects of (in)congruency on response times could be really reflected imitative motor responses or could be explained by more general stimulus-response incompatibility effects. The setup, explained in detail below, ensured maximal comparability with the SAMT. Additionally, we also conducted the SAMT as in Experiment 1, to replicate the results of Experiment 1 and to allow for within-subject comparisons of the Simon task and the SAMT. The SAMTs of Experiment 1 and 2 were structurally equivalent, which further allowed us to pool the samples of our two experiments to enhance our analytical power [51].

### Method

#### Participants

Sixty-four right-handed, White students (50 female, 14 male; mean age: 22.3 years, *SD* = 2.25) who received course credit for participation were recruited. One female participant had to be excluded from data analysis due to technical errors. Previous participation in experiment 1 was an exclusion criterion and thus none of the participants from experiment 1 participated in experiment 2.

### Materials

#### Simon task

Trials in this task consisted of two frames displaying the same happy and angry in- and out-group faces as in the SAMT of Experiment 1. Instead of a hand, a fixation cross was shown on the first frame in the lower middle part of the image (see Fig 1). In the second frame, a number cue (a Black “1” or “2” on a grey square, as in Experiment 1) was displayed to the right or to the left of the fixation cross, cueing a finger lifting movement of the index or middle finger. In congruent trials, the “1” was shown on the right side and the “2” on the left side, i.e., in congruency with the relative spatial position of the fingers to be lifted; in incongruent trials, the position of the cues was reversed. Subtracting response times of congruent trials from response times of incongruent trials thus allowed us to assess the combined facilitation and interference effects of this congruency manipulation (see Fig 2), as done for the mimicry effect in the SAMT.

Trial duration was matched with trial duration of the social-affective mimicry task with the two frames each displayed for 1,500 ms, and a fixed intertrial-interval of 1,500 ms, resulting in a trial length of 3,000 ms with a total of 10 trials per condition and cue. This resulted in 160 trials and task duration of approximately 12 minutes.

#### Social-affective mimicry task (SAMT)

Stimulus content and presentation were identical to Experiment 1. Timing in the SAMT was slightly different as in experiment 1. The timing used in experiment 2 was nevertheless the same as used in Rauchbauer et al. (2015) [22] and aimed to test the replicability of results with altered presentation time of the first and last stimulus frame. The first and the last stimulus frame were displayed for 1,500 ms, frames 2 and 3 were unaltered with their presentation time of 34 ms. Intertrial-interval was set to 1,500 ms. 10 trials per condition, congruency (congruent, incongruent and baseline) and per target cue were presented, as in the Simon Task, resulting in a total of 240 trials and a duration of about 18 minutes. The same procedure as in Experiment 1 was used to correct for outliers before conducting further inferential statistical analyses on both the Simon task and the SAMT.

**Procedure:** Upon arrival, participants were given an informed consent form including detailed instructions. Participants then completed practice trials of the Simon task before proceeding with the actual task. The Simon task was always presented first, as the experimental focus lay on this specific paradigm. Between the Simon task and the SAMT they filled in some online questionnaires unrelated to the task to wipe out attentional carry-over effects of the preceding task. Participants filled out the Interpersonal Reactivity Index (IRI) [60], the Emotion Contagion Scale [61], and the Bermond-Vorst Alexithymia questionnaire version B (BVAQ-B) [62]. Following the questionnaires, they were asked to put on the beige gloves and to perform the SAMT. After this they completed the threat-IAT (as in Experiment 1) and subsequently the Attitudes towards Blacks Scale [54].

### Results

In the Simon Task the winsorising procedure identified 10 datapoints as outliers with mean RTs higher than 75^th^ percentile plus 1.5 times the interquartile range of the conditions per target cue. These datapoints were thus replaced with the maximum mean RT in the specific condition. Additionally, 2 datapoints with mean RTs lower than 75^th^ percentile plus 1.5 times the interquartile range of the conditions per target cue were replaced with the minimum mean RT in the specific condition (together 7.5% outliers). In the SAMT the winsorising procedure identified 19 datapoints (7.92%) of the subjects’ individual mean RT per condition and target cue as outliers, with mean RTs higher than the 75^th^ percentile plus 1.5 times the interquartile range of the conditions per target cue. These datapoints were thus replaced with the maximum mean RT in the specific condition.

#### Simon task

The main focus of this analysis was to investigate the modulation of the Simon task by the social-affective cues. For comparability with the SAMT, the difference scores (incongruent—congruent trials) were entered into a two-way repeated measures ANOVA with factors Group (In-group, Out-group) and Emotion (Happy, Angry). This revealed a significant Group x Emotion interaction (*F*(1, 62) = 5.31, *p* = .025, partial η^2^ = .08). Planned comparisons (Bonferroni corrected for four comparisons significance level ≤ .0125) revealed a significantly lower difference in RTs in response to displays of happy in-group members than angry in-group members (*t*(62) = -2.79, *p* < .001, angry in-group: *M* = 70.58, *SE* = 4.65, happy in-group: *M* = 58.62, *SE* = 4.5). By trend presentation of Black happy faces elicited longer RTs than angry Black faces (*t*(62) = 1.96, *p* = .055, happy out-group: *M* = 72.427, *SE* = 3.92, angry out-group: *M* = 65, *SE* = 4.97).

Furthermore, mean RTs per condition were entered into a three-way repeated measures ANOVA with factors Group (In-group, Out-group), Emotion (Happy, Angry) and Congruency (Congruent, Incongruent). This revealed main effects for Group with higher RTs for Out-group, for Emotion with higher mean RTs for angry faces and for Congruency with higher mean RTs for incongruent trials (Group: F(1, 62) = 7.49, *p* = .008, partial η^2^ = .108; In-group: M = 488.16, SE = 6.083, Out-group: M = 492.704, SE = 6.361; Emotion: F(1, 62) = 252.028, *p* ≤ .001, partial η^2^ = .804; Happy: M = 473.754, SE = 6.002, Angry: M = 507.105, SE = 6.499; Congruency: F(1, 62) = 262.548, *p* ≤ .001, partial η^2^ = .809; Congruent: M = 474.07, SE = 6.154, Angry: M = 506.789, SE = 6.344). Results showed a significant interaction effect for Group x Emotion F(1, 62) = 249.126, *p* ≤ .001, partial η^2^ = .801), as well as for Group x Congruency F(1, 62) = 284.947, *p* ≤ .001, partial η^2^ = .821) and Group x Emotion x Congruency F(1, 62) = 5.31, *p* = .025, partial η^2^ = .079). As for the SAMT we carried out separate 2 x 2 repeated measures ANOVAs for the factors Group (In-group, Out-group) and Emotion (Happy, Angry) per Congruency (Congruent, Incongruent). For congruent trials this revealed a significant interaction for Group x Emotion (F(1, 62) = 7.686, *p* = .007, partial η^2^ = .079)). Planned comparison (Bonferroni corrected for four comparisons significance level ≤ .0125) by trend revealed higher mean RTs for angry Black faces than White ones (*t*(62) = 2.279, *p* = .026, angry out-group: *M* = 460.618, *SE* = 6.158, angry in-group: *M* = 451.853, *SE* = 6.124). Planned comparisons also by trend revealed higher mean RTs for White happy than angry faces (*t*(62) = 2.38, *p* = .020, happy in-group: *M* = 459.856, *SE* = 6.346). For incongruent trials results revealed a significant main effect for Emotion (F(1, 62) = 6.989, *p* = .01, partial η^2^ = .101) with higher mean RTs for angry than happy faces (angry: M = 527.456, SE = 7.242; happy: M = 520.456, SE = 6.544).

#### Social-affective mimicry task (SAMT)

**Mimicry effect:** We aimed to replicate and thus fortify the results of Experiment 1. Therefore, the values of the mimicry effect (difference measure) were entered into a two-way repeated measures ANOVA with factors Group (In-group, Out-group) and Emotion (Happy, Angry). This revealed a significant Group x Emotion interaction (*F*(1,60) = 5.378, *p* = .024, partial η^2^ = .08; see Fig 2). Paired *t*-tests (Bonferroni corrected for four comparisons significance level p ≤ .0125) showed, by trend, a higher mimicry response when angry out- than angry in-group faces were displayed (*t*(60) = 2.36, *p* = .022; see Table 1 for details). Participants’ mimicry effect was by trend also significantly higher when seeing angry than happy out-group faces (*t*(60) = 2.37, *p* = .021, see Table 1 for details). No other significant result was found (*p* ≥ .683 .05, see Table 1 for details).

**Response facilitation:** We were specifically interested in whether the modulation of the mimicry response was based on facilitation of mimicry. First we tested for effects of social-affective and congruency conditions. Therefore, mean RTs per condition were entered into a three-way repeated measures ANOVA with factors Group (In-group, Out-group), Emotion (Happy, Angry) and Congruency (Congruent, Incongruent, Baseline). This revealed a main effect of Congruency (*F*(2,120) = 153.917, *p* < .001, partial η^2^ = .7). Mean RTs on congruent trials were lower than for baseline (*t*(60) = -13.35, *p* < .001, congruent: *M* = 449.019, *SE* = 5.66, baseline: *M* = 476.214, *SE* = 6.13); and also lower for incongruent trials (*t*(60) = -14.23, *p* < .001, incongruent: *M* = 492.757, *SE* = 7.078), as expected. For the interaction effect of Group x Emotion x Congruency we observed a trend towards significance (*F*(2, 120) = 2.25, *p* = .11, partial η^2^ = .04).

According to our hypotheses we conducted further two-way repeated measures ANOVAs on the factors Group and Emotion separately for congruent trials. We found a trend significant interaction Group x Emotion (*F*(1,60) = 3.86, *p* = .054, partial η^2^ = .082). Planned comparisons revealed a trend significant effect in the expected direction (Bonferroni corrected for four comparisons significance level p ≤ .0125). Results revealed a trend in the direction of enhanced facilitation when presenting out-group versus in-group angry faces (*t*(60) = -1.93, *p* = .059, see Table 1 for details). Furthermore a significantly higher response facilitation was found during presentation of angry out-group faces, as compared to happy out-group faces (*t*(60) = -2.07, *p* = 0.043; see Table 1 for details). Further repeated measures ANOVAs on only the incongruent or the baseline trials with the factors Group x Emotion showed no significant differences (*p* ≥ .213; see S2 File for details).

#### Comparison of effects on Simon task and SAMT

We aimed to rule out the alternative explanation that our result of the SAMT could be attributed to domain general effects related to response conflict. As illustrated in Fig 2, the spatial compatibility effects in the Simon task showed a pattern that was markedly different from the one observed for mimicry effects in the SAMT. Focus of this analysis was to directly compare the difference scores of the two tasks (obtained by subtracting the mean RT on congruent from those on incongruent trials) using a three-way repeated measures ANOVA with factors Group (In-group, Out-group), Emotion (Happy, Angry), and Task (SAMT, Simon task). We found a significant main effect of Task (*F*(1,60) = 48.37, *p* < .001), as well as a Task x Group x Emotion interaction (*F*(1,60) = 9.81, *p* = .003). We explored this interaction in more detail (Bonferroni corrected for four comparisons significance level p ≤ .0125) and compared (separately for both tasks) angry: in- minus out-group with happy: in- minus out-group, as well as in-group: angry minus happy versus out-group: angry minus happy. This revealed significant differences between the tasks for happy in-group minus out-group conditions (*t*(60) = 2.58, *p* = .012; difference for SAMT: *M* = 1.00 ms, *SE* = 4.04; Simon-task: *M* = -14.85, *SE* = 5.03), as well as, by trend, for angry out-group minus in-group conditions (*t*(60) = 2.27, *p* = .027, SAMT: *M* = 11.46, *SE* = 4.9, Simon-task: *M* = -6.38, *SE* = 6.16). Similar effects were found when comparing the two emotions for out-group conditions (happy minus angry out-group: *t*(60) = -2.9, *p* = 0.006, difference mean SAMT out-group: *M* = -10.77, *SE* = 4.55, difference mean Simon-task out-group: *M* = 8.06, *SE* = 3.9). No other significant differences were found (all p’s ≥ .064).

In a four way repeated measures ANOVA we also compared the Simon task and the SAMT with the factors Group (In-group, Out-group), Emotion (Happy, Angry), Congruency (Congruent, Incongruent) and Task (SAMT, Simon task). This revealed a trend significant main effect of Group with faster mean RTs for In-Group faces (*F*(1,60) = 3.953, *p* = .051, partial η^2^ = .062; In-group: M = 479.671, SE = 5.972, Out-group: M = 482.062, SE = 5.921). Furthermore, we found a significant main effect for Congruency, with faster mean RTs on congruent trials as compared to incongruent ones (*F*(1,60) = 426.083, *p* < .001, partial η^2^ = .871; Congruent: M = 453.218, SE = 5.453, Incongruent: M = 508.514, SE = 6.622). Also we found a main effect for Task with faster mean RTs on the SAMT (*F*(1,60) = 22.504, *p* < .001, partial η^2^ = .273, SAMT: M = 470.881, SE = 6.223, Simon Task: M = 490.851, SE = 6.335). Results revealed significant interaction effects for Congruency x Task *F*(1,60) = 48.371, *p* < .001, partial η^2^ = .446), as well as for Group x Emotion x Congruency x Task (*F*(1,60) = 9.806, *p* = .003, partial η^2^ = .14)). In a next step we also calculated separate repeated measures ANOVA with the factors Group (In-group, Out-group), Emotion (Happy, Angry), and Task (SAMT, Simon task) per congruent and incongruent trials, to investigate whether response facilitation and inhibition were differentially affected in the two tasks. For congruent trials this revealed a trend significant result for Task with faster RTs for the SAMT (*F*(1,60) = 3.816, *p* = .001, partial η^2^ = .06; SAMT: M = 449, 019, SE = 5.663; Simon task: M = 457.418, SE = 6.053) and a significant interaction for Group x Emotion x Task (*F*(1,60) = 13.948, *p* < .001, partial η^2^ = .189). Planned comparisons (Bonferroni corrected for four comparisons significance level ≤ .0125) revealed a trend significant effect comparing mean RTs for the presentation of White happy faces (*t*(60) = 2.456, *p* = .017; SAMT: M = 449.157, SE = 6.239; Simon Task: M = 460.724, SE = 6.429). Also we found a significant difference for the presentation of Black angry faces (*t*(60) = 3.155, *p* = .003; SAMT: M = 461.125, SE = 6.312; Simon Task: M = 445.122, SE = 5.659). For incongruent trials results revealed a significant main effect for Group (*F*(1,60) = 5.839, *p* = .019, partial η^2^ = .089) with faster RTs for White than Black faces (In-group: M = 506, 136, SE = 6.506; Out-group: M = 510.892, SE = 6.879). Moreover results revealed a significant main effect for Task (*F*(1,60) = 44.229, *p* < .001, partial η^2^ = .424) with generally faster RTs in the SAMT than in the Simon task (SAMT: M = 492.744, SE = 7.078; Simon task: M = 534.285, SE = 6.99).

#### Correlation analysis Simon Task and SAMT

We performed Pearson correlation of the mean difference scores of the Simon Task and the SAMT per condition. This revealed significant correlation for both the presentation of in- and out-group happy faces (Happy: In-group: r = .304, p = .017; Out-group: r = .377, p = .003) in the absence of significant correlations for the respective presentation of angry faces (all ps ≥ .086).

#### Threat-IAT

Data of 3 participants had to be excluded due to erroneous performance on the task, resulting in data of 60 participants (46 female, 14 male; mean age: 22.26; *SD* = 2.26) entering data analysis. The same analysis procedure as in Experiment 1 was applied to the data. The *D*-measure showed a medium sized effect with *M* = 0.34, (*SE* = 0.35; see Table 2 for details), reflecting a stronger association between Blacks and threat than between Whites and threat. This replicated the result found in Experiment 1.

Pearson correlations on the mimicry effects per condition with the individual *D*-measure of the IAT revealed no significant correlations (all *r*-values ≤ 0.21; all *p-values* ≥ 0.11). We did not find any further significant correlations between the individual *D*-measure of participants and the differences of specific comparisons between reaction times when angry out-group versus in-group stimuli were shown, neither with the corresponding comparison when happy stimuli were shown between groups (all *r*-values ≤ -0.073; all *p-values* ≥ 0.58).

#### Attitudes towards black scale

Data of all 63 participants were included in the analysis. Participant´s explicit attitude towards Blacks, was, as in Experiment 1, neutral to favorable (*M* = 4.06, *SE* = 0.45). The mean score of the Attitudes towards Black Scale did not correlate with the size of the mimicry effect in the SAMT, nor the individual D-measure in the IAT (ATB and SAMT: all *r*-values ≤ 0.14; all *p-values* ≥ 0.29; ATB and D-measure: *r* = -0.15; *p* = 0.25).

### Combined analyses of pooled data from the SAMT in Experiment 1 and 2

#### Mimicry effect

To enhance the analytical power of our analyses, we pooled the original data of Experiments 1 and 2 (Experiment 1: *n* = 62; Experiment 2: *n* = 61) in a two-way repeated measures ANOVA with the factors Emotion (Happy, Angry) and Group (In-Group, Out-Group) treating Experiment (Experiment 1 and 2) as between-subject factor.

This revealed a significant Emotion x Group interaction (*F* (1, 121) = 12.84; *p* ≤ .001, partial η^2^ = .1), in the absence of further main and interaction effects (*p* ≤ .092). Since we did not reveal a significant difference between the mimicry effect of the two experiments, we performed planned comparisons on the combined sample (Bonferroni corrected for four comparisons significance level p ≤ .0125) (Experiment 1 and 2: *N* = 123). This revealed a significant difference of higher mimicry effect comparing the presentation of angry faces for in- and out-group (*t*(122) = 3.418, *p* = .001, see Table 1 for details). Also we found a trend significant difference the in-group happy condition with the in-group angry condition (*t*(122) = 2.5, *p* = .015, see Table 1 for details). Also planned comparisons showed a significant difference of higher mimicry effect when out-group angry compared to happy faces were presented (*t*(122) = -2.24, *p* = .011, see Table 1 for details). Results did not reveal significant differences in the mimicry effect when happy in- or out-group faces were presented (*p* = .139).

#### Response facilitation

We also performed a pooled three-way repeated measures ANOVA on participants mean reaction times with the within-subject factors Emotion (Happy, Angry), Group (In-, Out-Group), Congruency (congruent, incongruent, baseline), and Experiment (Experiment 1 and 2) as between-subjects factor. This revealed a significant main effect for Congruency, as well as Congruency x Experiment (Congruency: *F*(2, 242) = 284.2, *p* ≤ .001, partial η^2^ = .7, congruent: *M* = 457.28, *SE* = 4.97, incongruent: *M* = 511.03, *SE* = 6.18, baseline: *M* = 488.09, *SE* = 5.5) Congruency x Experiment: *F*(2, 242) = 9.23, *p* ≤ .001, partial η^2^ = .07). The results also showed a significant Group x Emotion interaction effect (*F*(1,121) = 2.27, *p* = 0.023, partial η^2^ = .042, In-group Happy: *M* = 485.22, *SE* = 5.42, In-group Angry: *M* = 486.01, *SE* = 5.55, Out-group Happy: *M* = 487.13, *SE* = 5.4, Out-group Angry: *M* = 483.49, *SE* = 5.57). We obtained a significant interaction effect of Group x Emotion x Congruency (*F*(2,242) = 5.77, *p* = .004, partial η^2^ = .05). To investigate the Congruency x Experiment interaction in more detail, we carried out planned comparisons of the different congruency conditions and experiment (Bonferroni corrected for four comparisons significance level ≤ .0125). This revealed significant differences for incongruent and by trend significant differences for baseline trials in the two experiments, with generally faster response times in Experiment 2 (Incongruent trials Experiment 1 versus Experiment 2: (*t*(59) = 2.98, *p* = .004; incongruent: Experiment 1: *M* = 528.08, *SE* = 10.3, Experiment 2: *M* = 491.77, *SE* = 7.129); Baseline Experiment 1 versus Experiment 2: (*t*(59) = 2.16, *p* = .0035; Baseline: Experiment 1: *M* = 499.266, *SE* = 9.35, Experiment 2: *M* = 475.628, *SE* = 6.205). Mean reaction times in the congruent trials did not differ between experiments (*p* = .091). Second, due to the three-way interaction of Group x Emotion x Congruency and to test our hypothesis that response facilitation was driving the modulation of the mimicry effect, we performed separate repeated measures ANOVAs for congruent, incongruent and baseline trials with the factors Emotion (Happy, Angry) and Group (In-, Out-Group). Even though we did not find an effect of Experiment on the three-way interaction of Group x Emotion x Congruency, considering that the SAMT in Experiment 1 and 2 differed in terms of presentation time and administration of the SAMT after the Simon task in Experiment 2 we included Experiment as a between-subject factor. We aimed to substantiate whether the effects on mimicry were based on response facilitation, as expected, on response interference, or general attentional or arousing effects due to the task-irrelevant social-affective stimulus quality in the bigger sample (Experiment 1: n = 62, Experiment 2: n = 61). For congruent trials this revealed a significant effect for the interaction Emotion x Group (*F*(1, 121) = 19.04; *p* ≤ .001, partial η^2^ = .14)in the absence of other significant results (*p ≥* .069). No significant effects were found for incongruent or baseline trials (*p* ≥ .085). Planned comparisons (Bonferroni corrected for four comparisons significance level ≤ .0125) for congruent trials showed a significant difference response facilitation when angry in- and out-group faces were presented, with faster reaction times on presentation of angry out-group faces (*t*(122) = -3.699, *p* ≤ .001, see Table 1 for details). A trend significant facilitation effect, was also found when presenting happy in- than out-group faces, with faster reaction times for happy in-group faces (*t*(122) = -2.367, *p* = .019, see Table 1 for details). As for same group membership, response was significantly facilitated (*t*(122) = -3.9, *p* ≤ .001) when out-group angry, rather than the respective happy face was presented. For the in-group we found a trend significant facilitative effect when displaying in-group happy faces, as compared to the respective angry face (*t*(122) = -2.4, *p* = .018). We also aimed to investigate this interaction in additional detail. (See S3 File for additional analysis of response inhibition and baseline trials)

### Discussion

In Experiment 2 we conducted an adapted version of the Simon task, to test whether the effects of our conditions on response times in the SAMT might have been driven by domain general processes related to (in)congruency and response conflict rather than being specifically related to an imitative motor response. In addition to testing this alternative hypothesis for the modulation of the found effect, we also aimed to replicate the results of Experiment 1 in a structurally equivalent SAMT, which also enabled us to pool data of Experiment 1 and 2 together to enhance the analytical power of our analysis and to further substantiate our hypotheses.

First, the spatial (in)congruency effects in the Simon task showed a diametrically opposed pattern than the effects observed in the SAMT, both for the difference measure, as for response facilitation. Moreover, the mimicry effect of happy in- and out-group faces correlated with the mean difference score of incongruent minus congruent trials in the Simon Task, yet not for the presentation of angry faces. Thus, the effects found in the latter cannot be explained by domain general (in)congruency effects but seem to be specific to a motor resonance process. Overall, the responses in the social-affective mimicry task were faster than in the first study, most likely due to training effects, as the Simon task was always presented first. Nevertheless, including Experiment as a between-subject factor into the repeated measures ANOVA, this revealed no effect of Experiment.

As in Experiment 1, the results from the threat-IAT show a medium effect of Black people being implicitly more associated with threat than White people, while no such association could be revealed with explicit measures (Attitudes towards Blacks Scale). This seems in line with the notion that the mimicry effects to angry out-group members are elicited by implicit attitudes towards Blacks, and not explicitly cognitively controlled.

Concerning our study aims, first we replicated the result for regulation of the mimicry effect in response to angry out-group members as compared to angry in-group members found in Experiment 1. Also combining the samples of Experiment 1 and 2 replicated this main finding and thus corroborates the appeasement hypothesis.

Experiment 2 and the pooled analysis also show a significant difference between happy and angry out-group faces, but no difference for this comparison in the in-group condition. Directly comparing the mimicry response to in-group members does not yield a difference and this might suggest that mutual group-membership may in itself elicit a wish for affiliation (e.g. [35, 40].

Yet, we did not replicate the difference in the mimicry effect to happy in-group vs. happy out-group faces, also not when increasing statistical power by combining the samples of experiments 1 and 2 (*N* = 123). As mentioned before, these findings are in line with reports from facial mimicry studies, where out-group happy faces were found to be mimicked in a similar way as in-group happy faces [10]. Yet our findings extend it to mimicry of arbitrary finger lifting movements, i.e. movements which are not carrying intrinsic affective information. This may be related to the fact that happiness signals cooperation and prosocial motivation from the expresser [42], which may explain why it is equally responded to with an implicit affiliation signal for both in- and out-group members [10]. Thus, reciprocation of affiliation via mimicry might take place regardless of group-membership.

Finally, for the response facilitation effect we found a trend significant result in Experiment 2 for enhanced response facilitation in congruent trials driving the mimicry effect. Nevertheless, the significant result when combining both samples (*N* = 123), thus enhancing analytical power, confirms our hypothesis that the adaptive regulation of the mimicry effect tailored to affiliative goals is driven by enhanced response facilitation on congruent trials.

## Experiment 3

Experiments 1 and 2 demonstrated an enhanced mimicry response towards angry out-group members, which is suggestive of appeasement attempts of a threatening other. Experiment 2 bolstered this interpretation by showing that the results are specifically related to mimicry and not to a domain-general (in)congruency confound. Based upon these findings we aimed to provide more direct evidence that it was indeed the potentially physically threatening attributes of the angry out-group stimuli that were driving the effects on mimicry. Thus Experiment 3 varied the amount of threat posed by angry in- and out-group members via vignettes. We predicted that a higher amount of personal threat posed by an angry out-group member would elicit an enhanced mimicry effect, and that this would furthermore be driven by response facilitation in congruent trials.

### Method

#### Participants

Fifty-eight right-handed, White, German-speaking students from the University of Vienna participated in exchange for course credit (38 female, 21 male; mean age: 24.22 years, *SD* = 6.45). Previous participation in experiment 1 and 2 was an exclusion criterion and thus none of the participants from experiment 1 or 2 participated in experiment 3.

#### SAMT with vignettes

**Stimuli and experimental design:** In order to examine whether the amount of personal threat experienced from an in- or out-group member influences the mimicry effect, vignettes were presented to frame angry in- and out-group faces as threatening or not threatening towards other persons. In the Personal Threat condition, the individuals showing angry expressions were described as unpredictably physically aggressive, for instance because they had physically attacked a stranger in the subway for no particular reason. In the No Personal Threat condition, the person in the vignette was described as an environmental enthusiast attending pro-environmental demonstrations, and that in this context she had crashed a window once, but would never harm a person. We added the No Personal Threat condition as a plausible explanation for why the shown person expressed anger, but that this anger does not represent a personal threat, in contrast to the anger expressed by the potentially physically aggressive person shown in the Personal Threat condition. We also used happy faces in Experiment 3 to maximize comparability between the three experiments, and to avoid confounds such as different anchor or habituation effects when only presenting angry faces. For happy faces we presented a vignette in which activities the person enjoyed were described. However, happy faces were not analyzed as our main focus was on the vignette effects of personal vs. non personal threat (but see analysis including happy faces in S4 File). A different female face was used for every condition. The vignettes were matched for content per condition across groups and text length; assignment of condition and vignettes to face stimuli was counterbalanced across participants. Stimulus presentation for the SAMT was identical to Experiment 1.

The experimental design was a 2 x 3 x 2 design with factors Group (In-group, Out-group), Threat (Happy, Angry/Personal Threat, Angry/No Personal Threat) and Congruency (Congruent, Incongruent). Trials were presented in blocks of five trials per condition (e.g., five trials of Angry In-group / Personal Threat); each block was presented twice resulting in a total of 10 trials per condition and target cue (240 trials in grand total). Order of blocks was pseudo-randomized, ensuring that identical blocks were not presented consecutively. Each block started with a depiction of a female face with the corresponding vignette (see Fig 1). Timing of stimulus presentation matched the timing of Experiment 1 with the first frame presented for 2,000 ms, followed by consecutive presentation of frame 2 and 3 for 34 ms and the last frame for another 1,232 ms, resulting in a trial length of 3,300 ms, inter-trial interval set at 2,700 ms. This resulted in 240 trials in total and a duration of approximately 25 minutes (exclusive free reading time for vignettes). The same winsorising procedure as in Experiments 1 and 2 was applied to remove outliers from the data. The mimicry effect was, as in the previous experiments, calculated as the difference of mean RTs of incongruent minus congruent trials per social-affective condition.

**Procedure:** Participants were given a detailed informed consent form including instructions about the task. After this, they were asked to put on the beige gloves and completed several practice trials of the mimicry task without the vignettes. They were then instructed about the actual task: they were asked to read the vignettes carefully, to imagine the person in the described situation, and to keep a vivid image of her in their minds while performing the subsequent task. As soon as they had memorized the description they could proceed with the task self-paced, by pressing a key. After finishing the mimicry task participants were asked to perform the threat-IAT and to fill out the Attitudes towards Blacks Scale.

### Results

#### SAMT with vignettes

The winsorising procedure identified 23 datapoints (12.78%) of the subjects’ individual mean RT per condition and target cue for the factors Group (In-group, Out-group) and Threat (Angry/Personal Threat, Angry/No Personal Threat) per middle and index finger (180 trials in total) as outliers with mean RTs higher than the 75^th^ percentile plus 1.5 times the interquartile range of the conditions per target cue. These datapoints were thus replaced with the maximum mean RT in the specific condition. (Note: 10 datapoints (43%) of the 23 outliers were identified in the White Angry/Personal Threat condition, 5 datapoints (21.7% of the outliers) in the Black Angry/Personal Threat condition).

**Mimicry effect:** The aim of this analysis was to substantiate our main finding of Experiments 1 and 2, that enhanced mimicry towards angry out-group members was specifically driven by perceived personal threat. We performed a repeated measures ANOVA with factors Group (In-group, Out-group) and Threat (Angry/Personal Threat, Angry/No Personal Threat) on the mimicry effect (i.e. difference measure of mean RT incongruent—congruent trials) (see Fig 2). This revealed a significant Group x Threat interaction (F(1,57) = 6.95, *p* = .011, partial η^2^ = .11). The results are displayed in Fig 2. We specifically expected the mimicry effect to differ across groups in the Personal Threat condition. We carried out planned comparisons (Bonferroni corrected for four comparisons significance level p ≤ .0125), which fortified this hypothesis and showed a significantly stronger mimicry effect when Out-group / Personal Threat stimuli were presented than In-group Personal Threat stimuli (*t*(57) = 22.76, *p* = .008, see Table 1 for details). Moreover, the effect of type of threat was different for the In-group, as shown by a significant difference when In-group / No Personal Threat was compared to In-group / Personal Threat, with a higher mimicry effect for In-group / No Personal Threat (*t*(57) = 3.34, *p* = .001, see Table 1 for details). No other significant differences were found (all p’s ≥.303).

**Response facilitation:** The focus of this analysis was to fortify our hypothesis that the modulation of the mimicry effect between the Personal Threat vs. the No Personal Threat condition was driven by response facilitation. We first performed a three-way repeated measures ANOVA with the factors Threat (Personal Threat/ No Personal Threat), Group (In-, Out-Group) and Congruency (Congruent, Incongruent). This revealed a main effect of Congruency (*F*(1,57) = 396.84, *p* ≤ .001, partial η^2^ = .87; Congruent: *M* = 474.86, *SE* = 8.62, Incongruent: *M* = 544.88, *SE* = 9.37). Results also revealed a significant interaction effect of Threat x Group x Congruency (*F*(1,57) = 6.95, *p* = .011, partial η^2^ = .11) Second, we specifically investigated whether this interaction effect was due to response facilitation on congruent trials. We performed a 2 x 2 (Group x Threat) repeated measures ANOVA for both congruent and incongruent trials separately. This revealed a significant Group x Threat interaction effect of response facilitation on congruent trials (*F*(1,57) = 5.97, *p* = .018, partial η^2^ = .10; see Table 1 for details). Exploration of this interaction with planned comparisons (Bonferroni corrected for four comparisons significance level p ≤ .0125) revealed a trend towards a higher facilitation effect (i.e., faster reaction times) when angry out-group faces were presented threatening to physical safety than when they were presented as a non-threatening (*t*(57) = 1.93, *p* = .058). Planned comparisons also, by trend, showed a higher mimicry effect when angry in-group faces were presented as non-threatening as compared to threatening (*t*(57) = -2.11, *p* = .039). No differences were found when both in- and out-group were presented as threatening (*p* = .083), respectively non-threatening to physical safety (*p* = .115).

**Response Inhibition:** We found a trend significant result for the 2 x 2 (Group x Threat) repeated measures ANOVA for incongruent trials with a trend significant result for Threat (*F*(1,57) = 3.73, *p* = .058, partial η^2^ = .06) with slower reaction times for the presentation of no personal threat (No Threat: M = 548.8, SE = 9.54; Personal Threat: M = 540.96, SE = 9.63). No other significant results were found (all ps ≥ .41).

#### Threat-IAT

Data from 51 participants (34 female, 17 male; mean age: 22.46 years, *SD* = 2.6) entered data analysis; data from eight further participants had to be excluded from the analysis, due to erroneous responses or technical errors. Preprocessing of data was carried out as in Experiments 1 and 2. The analysis revealed a mean *D*-effect of *M* = 0.51 (*SE* = 0.038; see Table 2 for details), indicating a medium to large effect of associating Black people more with threat than White people. Pearson correlation of the mimicry effects (i.e., difference measure of incongruent—congruent trials) per condition, as well as the difference measures of the specific comparisons with the individual *D*-measure of every subject in the IAT revealed no significant results (all *r*-*values* ≤ 0.10; all *p-values* ≥ 0.50).

#### Attitudes towards blacks scale

Data of 57 participants was included in the data analysis; due to technical errors one female could not finish the questionnaires and was excluded from this analysis. As in Experiment 1 and 2, participants showed a neutral explicit attitude towards Blacks, (*M* = 4.07, *SE* = 0.05). Pearson correlation with the individual mean scores in the Attitudes towards Black Scale revealed no significant Pearson correlations (all *r*-*values* ≤ 0.10; all *p-values* ≥ 0.45).

### Discussion

Experiment 3 revealed that angry out-group members perceived as potentially threatening elicit a stronger mimicry effect in arbitrary finger lifting movements than personally threatening angry in-group members. There was no such difference between angry in-group and out-group members who were framed as not personally threatening. This replicates our main finding of Experiment 1 and 2 and substantiates that the perception of an angry out-group member as potentially physically threatening may have driven the effects. A Black out-group member perceived as personally threatening might elicit higher mimicry, and this would be in line with mimicry’s function as an affiliative signal (e.g. [3, 7, 8, 9]), which in this case serves to prevent conflict via appeasement. Note also that mimicry has been shown to increase empathy towards out-groups [17]. This underlines that mimicry in potentially threatening siutations with an out-group member may indeed have apositive and potential de-escalting function. Furthermore, Experiment 3 extends these results by suggesting that, when dealing with a mutual group member, who is personally threatenening, mimicry is enhanced as compared to a non-threatening, but angry, mutual group member. This suggests that mimicry may be an affiliative signal used also for appeasment in an in-group context, in the absence of personally threatening out-group members. In a social context specified as not personally threatening, the mimicry response is not enhanced, also regardless of group membership. In this case, the need to soothe a conflict by appeasement may not be perceived as vital. As such these results confine enhanced mimicry to personal threatening situations. The absence of a difference between mimicry towards angry out-group members both personally threatening or not, in the absence of comparison to an in-group member, might mirror the general effect of angry out-group members implicitly perceived as threatening.

For response faciliation’s proposed social signalling function though we found faster responses when both In- and Out-group members were presented as threatening. This was accompanied by a trend of less inhibition for the presentation of personal threat. As such experiment 3 adds valubale information to the social signalling function of response congruency in personally threatening situations. Enriching the social context with specific personal threat information first, seems to, enhance response times in threatening situations and lower inhibition. Second, it also shows, that in explicitly framing the context as personally threatening both In- and Out-group members elicit higher repsonse faciliation.

Experiment 3 also replicated the results of the threat-IAT found in the two previously reported experiments. This again revealed a medium effect of Black people being implicitly more associated with threat than with security. In the Attitudes towards Blacks Scale participants exhibited, as demonstrated in Experiments 1 and 2, a neutral to favorable attitude towards Blacks in the corresponding questionnaire. This replicates once more that the regulation of the mimicry effect happened unconsciously.

Overall, the results of experiment 3 replicate the main consistent finding of the paper, that angry out-group members, who may be perceived as personally threatening, elicit a higher mimicry response than personally threatening in-group members.

## General Discussion

The purpose of the present study was to assess the malleability of mimicry of arbitrary finger lifting movements by affiliation-related social-affective cues. The main aim was to identify an affiliative function of mimicry that has thus far been overlooked: to appease a potentially threatening other. Thus, we examined whether the regulation of mimicry in response to such cues might be not only tailored to reciprocation of affiliation, but also to appease an unfavorable social interaction. Furthermore, we aimed to elucidate whether this tailored malleability of mimicry was based on response facilitation (of congruent movements) or response interference (with incongruent movements). In three experiments we conducted the social-affective mimicry task (SAMT), in which we simultaneously presented, in addition to the task-irrelevant hand stimulus, facial stimuli of females from either the own or another ethnic group with either happy or angry facial expressions. Thus, according to our main aim, the most consistent finding of the present study, established in the first and replicated across all three experiments, is increased mimicry of finger lifting movements when angry out-group faces were presented (as compared to angry in-group faces). This effect seems to be confined to (angry out- vs. in-group) stimuli framed as potentially posing a physical threat to oneself, as shown in Experiment 3.

In the absence of correlations, the threat-IAT nevertheless, across all three experiments consistently revealed that Black (compared to White) out-group members were implicitly moderately associated with threat. Furthermore, the results of Experiment 2 suggest that the results of the SAMT were not driven by a domain general process of cognitive response conflict but were in fact based upon motor resonance processes. Furthermore, we also consistently found that the social-affective stimuli modulated response facilitation (i.e. congruent trials), potentially signaling social congruency. In experiment 3 we also found that the specific context of Personal threat may also have also influenced response inhibition.

The present study suggests that the results cannot solely be explained by generalized changes in arousal, attention or enhanced efficiency in memory encoding [57]. If this would have been the case, it should have been reflected in generally faster response times across all conditions in response to the stimuli with highest salience, i.e. the angry out-group faces. Let alone in Experiment 3, we found that the framing personal threat was by trend lowering response inhibition. Yet, this result does not challenge the suggestion, and consistent result, that mimicry might also have an appeasement function. It highlights that the vignettes might have indeed created a realistic social context of personal threat, accompanied by enhanced attention to a salient social context. Considering the potential practical importance of the “mimicry for appeasement” hypothesis, it is highly suggested to test this result in a natural social interaction. Nevertheless, in experiment 1 and 2, as well as the pooled data, we did not observe significant modulations across conditions in incongruent or baseline trials of the SAMT. Moreover, responses in the Simon Task were differently modified by angry out-group faces than in the mimicry task, which goes against the notion that these effects are driven by non-imitative attentional effects caused by the saliency of these stimuli. Thus, our findings seem to reflect a genuine imitative motor response that surpasses domain-general processes of response conflict, as well as general arousal and attention effects. Angry out-group faces might represent a highly salient social stimulus, which enhances mimicry of arbitrary movements as an affiliative signal in congruent trials.

### Regulation of Mimicry for Appeasement

Our main finding, which is also the most consistent one across all three experiments, is that counter-affiliative (i.e., angry) facial expressions increase mimicry when they are conveyed by out-group as compared to in-group members. This finding highlights a possible additional function of behavioral mimicry that has thus far received little attention: namely to implicitly appease a threatening interaction partner, and to ease a potential conflict. That is, the stimuli of out-group members that conveyed counter-affiliative intent and potentially appeared aggressive might have been experienced as threatening. We propose that implicitly perceived threat posed by an out-group member, leads to a regulation of mimicry for appeasement purposes, which was confirmed by Experiment 3. It has been shown that being mimicked leads to enhanced empathy towards out-group members [17]. This underlines the suggested positive effects of mimicry on the mimicked out-group member and may demonstrate that displaying mimicry may indeed enhance positive attitudes towards the mimicking individual. This would be in line with our proposed function of mimicry for appeasement. Implications of this finding for intergroup relations should be considered and investigated. Findings of enhanced mimicry in response to negatively valenced pictures with non-social content have also been interpreted as representing a fight- or flight response [20]. Our results extend these findings by suggesting that the implicit perception of an out-group as potentially threatening (which carries negative valence), may also evoke enhanced mimicry for appeasement. This is also in line with recent findings by Rauchbauer and colleagues [22] revealing distinct neural processes underlying enhanced mimicry towards out-group members (presumably reflecting mimicry for appeasement) than towards in-group members (presumably reflecting mimicry for reciprocating affiliative intent). However, the present evidence and the findings of the fMRI study also showed some slight differences, as in the latter higher mimicry was found in response to only out-group members in general, and not only to angry ones. This may be explained by effects of the scanner environment, such as increased stress or a more constrained body position.

Angry in-group members, on the other hand, elicited decreased mimicry, even when framed as potentially physically threatening to the self. In-group members were implicitly more associated with security than threat (according to the results of the threat-IAT). The mutual group-membership and more experience with (also angry) members of the own ethnic group might dampen the impression of threat in comparison to an out-group member. Individuals might be more exposed to angry fellow group members and thus used to angry in-group faces. As such, an in-group member’s anger might be more predictable and thus not evoke appeasement attempts. Decreased mimicry in response to angry in-group members might express a withdrawal of affiliative displays. As such, the withholding of mimicry might indicate a first step towards exclusion of the angry in-group member from further beneficial in-group exchange, especially when they are posing a potential personal threat to another mutual group member (see also [50] for the process of stigmatization). The “black sheep effect” [63], which proposes that disliked in-group members may elicit more extreme negative evaluations than disliked out-group members, also seems in line with this. Yet, since we did not test this hypothesis specifically, this interpretation stays speculative and requires further testing.

The results also show that, with the exception of experiment 1 (but see the results of the combined sample of Experiment 1 and 2 and S4 File for results of presentation of happy faces in Experiment 3), the presentation of happy faces leads to the same mimicry response for both in- and out-group members. As suggested by Bourgeois & Hess (2008) [10] mimicry may be equally enhanced towards members of in- and out-group when they smile. This strongly suggest that reciprocation of affiliative signals via mimicry seemingly occurs regardless of group membership, which is in line with reports from studies on facial mimicry [10].

### Response Facilitation in Congruent Trials

Our results show that the regulation of mimicry seems to specifically affect response facilitation, as previously also found for direct eye contact [19]. Only in experiment 3, in which we explicitly framed the social context as personally threatening did we find a trend for diminished response inhibition, potentially due to the enhanced salience of the social framing. Opposed to our and Wang and colleagues’ [19] results, the modulation of the mimicry effect has recently been found to be driven by the interference effect (i.e., incongruent trials) when administering oxytocin [64]. Yet De Coster et al.´s (2014) [64] study lacked social feedback signals, such as ethnic faces and emotional expressions (in our study) or eye-contact [19]. Thus the social context of our and Wang´s modulation might have caused the effect on congruent trials, thus suggesting response facilitation of mimicry as a social congruency signal to respond to social cues, such as eye contact: On a process-level we consider response facilitation to represent a social feedback signal of enhanced congruency with an interaction partner. We propose that the social-affective cues in our experimental design evoked and conveyed affiliative goals. Thus, response facilitation might capture a social signaling process, enhancing mutual congruency. Yet the trend significant result in experiment 3 showing decreased response inhibition for framing the situation as personally threatening shall not be overlooked. This demonstrates that salient social contexts may decrease response inhibition for the sake of heightened attention. Yet the specific circumstances that affect response facilitation or response interference in mimicry paradigms, potentially entailing different underlying processes and functions, should be further elucidated. The present paper highlights that, by additional framing of the context as personally threatening or not, attentional factors might become relevant and decrease inhibition of motor resonance processes.

This question might also be embedded in the more general question on how mimicry is modulated by social context. According to Heyes (2011) [6] two potential modulatory mechanisms emerge: first, it is suggested that heightened attention to task-irrelevant social-affective stimuli would modulate the perceptive input of a motor action, and thus lead to more mimicry (i.e., input modulation). On the other hand, the behavioral output could be altered, leading to enhanced mimicry in overt behavior due to social context signals. Based on the findings from all 3 experiments, as well as the fact that we do not find a modulation of RTs in incongruent and baseline SAMT trials in experiments 1 and 2, it seems less plausible that our mimicry effects are driven by modulation of perceptual input by our face stimuli, in terms of output modulation. Results from experiment 3 rather suggest that the effective social context framing may add attentional aspects for a better reaction to the environment. As such, presenting the social-affective stimuli without extra social context information may lead to modulation of response facilitation as an immediate social feedback signal. Adding salient context information also may seem to release inhibition processes, potentially to enhance the signaling process, which seems in line with input modulation. As such, our experiment suggests, that according to the extent of information available in the social environment, both in- and out-put modulation may shape the mimicry response [6]. An alternative explanation for the selective effects on RTs on congruent trials comes from Kleiman et al. (2014) [65]: Incongruent trials might demand higher cognitive control to execute the task-relevant response, which might override the modulation of RT by the (salience of) social-affective stimuli. Yet, the findings from the Simon task in Experiment 2 seem to rule out that the observed mimicry effects were driven by such domain-general (in)congruency effects and instead suggest that they represent a genuine motor resonance process. This furthermore suggests that, in experiment 3 cognitive control was diminished in favor for effective responding. Nevertheless, our interpretation of the processes underlying the mimicry effects remains highly speculative and should be addressed in future studies. The investigation of the evolvement of the time course of underlying neural processes (e.g., with electroencephalography; EEG) generating the social-affective modulation of the mimicry effect could contribute considerably to our knowledge of how the modulation of mimicry is generated. Respectively it could elucidate if saliency influences the modulation of the mimicry response and when or how congruent and incongruent trials are separately modulated by social-affective stimuli in their neural dynamics over time. Also varying the explicit social context in which stimuli are presented may shed more light onto when attentional processes are mostly engaged in shaping the mimicry response. Automatic imitation paradigms as laboratory measures of mimicry would allow systematic investigation with neuroscientific methods, tapping into the neural processes generating mimicry´s modulation by social-affective cues.

### Limitations

Across all three experiments we chose an approach combining implicit and explicit measures assessing ethnic bias to substantiate our hypotheses about the implicit modulation of mimicry by the social cue of group-membership. This approach allowed us to disentangle implicit from explicit motivations modulating the mimicry effect. We did not find a correlation between the implicit measure of the threat IAT and the size of the mimicry effects in the SAMT. Nevertheless, the results of the presented threat IAT are in line with previous literature investigating the implicit perception of Blacks as threatening (e.g. [37, 39]) and indicate that out-group members are perceived as more threatening than in-group members in all three experiments. Yet, the absent correlations between IAT scores and mimicry effects does neither provide direct support for the mimicry-for-appeasement-hypothesis, nor for any alternative explanations. We speculate that the lack of correlations may also be due to the fact that the classical version of the IAT, as used here, might not have been perfectly tailored to detect any associations, as it combines the dimensions of threat/security and White/Black, rather than disentangling them. Future research might thus want to use an implicit measure that associated more directly the perception of angry Black faces with threat. Moreover, we did not include a measure investigating the hypothesized implicit perception of Whites with security. This would be a valuable extension to the present paradigm and could be included in further studies.

In the present study, we present a novel view that extends the potential functions of mimicry by suggesting that in unfavorable social situations mimicry might serve as an appeasement signal. This proposition awaits direct testing “out in the wild”, respectively in a naturalistic social (laboratory) environment. By presenting this extended view on mimicry’s affiliative functions, we highly encourage fortification of our hypotheses in a social mimicry setup (as e.g. used by [1, 8, 9, 11, 13]). This could entail exploration of participants’ motives to mimic, as well as controlling for other potential modulators of mimicry, such as the desire of White people to appear unprejudiced against Black individuals (e.g. [66]). Nevertheless, Experiment 3 provides compelling evidence that framing Black angry faces as personally threatening modulates mimicry in the predicted direction. Mimicking out-group members has been shown to reduce prejudice [16] and to have positive effects on empathy towards an out-group [17]. Thus, future studies might take our suggestion as a starting point to investigate whether mimicry could support the soothing of potential (inter-)group or interpersonal conflict. Furthermore, future studies may aim to take into account first, more diverse ethnic populations of participants (the present study consisted of only White participants) and/or stimuli to better separate effects related to race and group status; second may extend their study design to investigate potential positive effects of mimicry on both interaction partners. It is pointed out that the results of the SAMT show low effect sizes, which may have been expected, given the implemented subtle manipulation of the mimicry effect by the social-affective variables. Nevertheless, using the SAMT, we have suggested that mimicry may have an affiliative function for appeasement. This extends current views on mimicry’s affiliative functions and could potentially have practical implications for intergroup relations. As such, the SAMT lays important groundwork for further investigation of mimicry’s appeasement function.

Notwithstanding these cautions, in a series of three studies, as well as pooled analysis for enhanced statistical power (see [52] for best practice recommendations in social psychology) we not only replicated our main findings, but were also able to rule out alternative explanations of our effect. We chose to pool the data of experiment 1 and 2, despite the differences in their timeline and despite the presentation of the Simon task before the SAMT in experiment 2 for the sake of enhanced analytical power. To account for the potential differences in the experiments we incorporated the factor experiment as a between-subject factor, thus controlling for potential differences. We believe that pooling the data strengthens our main result that mimicry is enhanced in response to the presentation of angry out-group members.

## Conclusions

This study contributes to conceptual models of mimicry by suggesting that it may bear a regulative function to appease potentially threatening interactions with the goal to de-escalate a potential conflict [49]. This may be in line with observations of submissive and conciliatory behavior in other primates [47, 48]. Positive effects of mimicry on the reduction of racial prejudice [16] and increase in empathy towards out-groups have been demonstrated [17]. As such, affiliative signals in the form of behavioral mimicry might serve to soothe potential conflicts by eliciting positive attitudes in the opponent. Furthermore we found, that mimicry may be a means of reciprocation of an affiliative signal, such as a smile, regardless of group-membership. This finding is in line with a function of mimicry as “social glue” [3]. Regardless of the appearance of an enhanced mimicry effect as a uniform RT measure (a number) in the SAMT, it has been shown that distinct neural processes guide this flexible regulation of mimicry towards the distinct affiliative goals [22]. Second, the present work suggests that the adaptive regulation of mimicry is driven by a facilitative motor resonance effect. As outlined above, response facilitation might resemble a social signaling process of enhanced congruency with the other. Enhanced congruency might facilitate the affiliative signaling process of mimicry. Response inhibition may become relevant when enhanced saliency of a social context calls for heightened attention.

This study presents a suggestion for extension of social cognition models of mimicry’s affiliative function from the prominent and often cited role as an affiliative signal for social bonding [7] to a largely overlooked affiliative function of de-escalation by displaying affiliative signals. Our findings thus indicate that automatic imitation paradigms are well suited for studying the determinants of behavioral mimicry in a strictly controlled laboratory setting. Combining a classic automatic imitation paradigm with social-affiliative context information seems useful in bridging the gap between socially meaningful, but weakly controlled, naturalistic settings and well controlled, but often context-free, experimental paradigms. One specific advantage is that they allow shedding light on the differential involvement of processes such as response facilitation or response inhibition in the regulation of mimicry. Future studies should test this proposed model of mimicry as representing an appeasement signal. More direct evidence of mimicry’s potential appeasement function by using naturalistic social-psychological investigations may fortify mimicry as an interactional tool to ameliorate intergroup conflict.

Overall, the present study provides an extended view on the driving forces behind mimicry as a regulative affiliative signal. Via its laboratory model of automatic imitation, our findings highlight mimicry´s role as a social catalyst not only to subtly further glue together harmonizing interaction partners, but also to smother and appease potentially explosive encounters with threatening individuals.

## Supporting Information

S1 File Additional results experiment 1. Response inhibition and baseline trials (SAMT).
(PDF)

S2 File Additional results experiment 2. Response inhibition and baseline trials (SAMT).
(PDF)

S3 File Additional results pooled analysis experiments 1 & 2. Response inhibition and baseline trials (SAMT).
(PDF)

S4 File Additional results experiment 3. Mimicry effect, Response Facilitation and Inhibition including the factor *Happy* (SAMT with vignettes).
(PDF)
