# Correction: The Modulation of Mimicry by Ethnic Group-Membership and Emotional Expressions

**DOI:** 10.1371/journal.pone.0162935

**Published:** 2016-09-08

**Authors:** Birgit Rauchbauer, Jasminka Majdandžić, Stefan Stieger, Claus Lamm

The following information is missing from the Funding section: This article was supported by the Open Access Publishing Fund of the University of Vienna. The publisher apologizes for the error.

## References

[1] Rauchbauer B, Majdandžić J, Stieger S, Lamm C (2016) The Modulation of Mimicry by Ethnic Group-Membership and Emotional Expressions. PLoS ONE 11(8): e0161064 doi:10.1371/journal.pone.0161064 27557135 10.1371/journal.pone.0161064 PMC4996423

