# Supplementary material for: The Modulation of Mimicry by Ethnic Group-Membership and Emotional Expressions
Source: PLoS One. 2016 Aug 24;11(8):e0161064. doi: 10.1371/journal.pone.0161064 (PMC4996423; doi:10.1371/journal.pone.0161064)
Supplement: S1 File — Response inhibition and baseline trials (SAMT). (PDF) [file pone.0161064.s001.pdf]

S1 File

Additional results experiment 1

Response inhibition and baseline trials (SAMT)

We performed repeated measures ANOVAs separate on incongruent and baseline trials with the factors EMOTION (Happy, Angry), GROUP (In-, Out-Group). For incongruent trials, the measure of response inhibition, results revealed no significant main effect for the factor Group ($F(1,60) = .735$, $p = .395$, partial $\eta 2 = .012$), Emotion ($F(1,60) = .104$, $p = .748$, partial $\eta 2 = .002$) or the interaction effect Group x Emotion ($F(1,60) = .006$, $p = .937$, partial $\eta 2 < .001$). For baseline trials, this revealed no significant main effect for the factor Group ($F(1,60) = .404$, $p = .527$, partial $\eta 2 = .007$)), Emotion ($F(1,60) = .625$, $p = .432$, partial $\eta 2 = .010$) or the interaction effect Group x Emotion ($F(1,60) = 2.17$, $p = .146$, partial $\eta 2 = .034$).
