# Supplementary material for: The Modulation of Mimicry by Ethnic Group-Membership and Emotional Expressions
Source: PLoS One. 2016 Aug 24;11(8):e0161064. doi: 10.1371/journal.pone.0161064 (PMC4996423; doi:10.1371/journal.pone.0161064)
Supplement: S2 File — Response inhibition and baseline trials (SAMT). (PDF) [file pone.0161064.s002.pdf]

S2 File

Additional results experiment 2

Response inhibition and baseline trials (SAMT)

We performed repeated measures ANOVAs separate on incongruent and baseline trials EMOTION (Happy, Angry), GROUP (In-, Out-Group). For incongruent trials, the measure of response inhibition, results revealed no significant main effect for the factor Group ($F(1,60) = 1.195$, $p = .279$, partial $\eta 2 = .02$), Emotion ($F(1,60) = 1.103$, $p = .298$, partial $\eta 2 = .018$) or the interaction effect Group x Emotion ($F(1,60) = 1.586$, $p = .213$, partial $\eta 2 = .026$). For baseline trials, this revealed no significant main effect for the factor Group ($F(1,60) = .735$, $p = .395$, partial $\eta 2 = .012$)), Emotion ($F(1,60) = .104$, $p = .748$, partial $\eta 2 = .002$) or the interaction effect Group x Emotion ($F(1,60) = .006$, $p = .937$, partial $\eta 2 < .001$).
