# Supplementary material for: The Modulation of Mimicry by Ethnic Group-Membership and Emotional Expressions
Source: PLoS One. 2016 Aug 24;11(8):e0161064. doi: 10.1371/journal.pone.0161064 (PMC4996423; doi:10.1371/journal.pone.0161064)
Supplement: S3 File — Response inhibition and baseline trials (SAMT). (PDF) [file pone.0161064.s003.pdf]

S3 File

Additional results pooled analysis experiments 1 & 2

Response inhibition and baseline trials (SAMT)

We performed repeated measures ANOVAs separate on incongruent and baseline trials with the factors Emotion (Happy, Angry), Group (In-, Out-Group) as within-subject, and Experiment (1, 2) as between-subject factor. For incongruent trials, the measure of response inhibition, results revealed no significant main effect for the factor Group ($F(1,121) = 1.991$, $p = .161$, partial $\eta 2 = .016$), Group x Experiment ($F(1,121) = .527$, $p = .469$, partial $\eta 2 = .004$), Emotion ($F(1,121) = .435$, $p = .511$, partial $\eta 2 = .004$) or Emotion x Experiment ($F(1,121) = 3.013$, $p = .085$, partial $\eta 2 = .024$). Results further did not show any significant interaction for Group x Emotion ($F(1,121) = 1.222$, $p = .271$, partial $\eta 2 = .010$) or Group x Emotion x Experiment ($F(1,121) = .032$, $p = .858$, partial $\eta 2 < .001$). For baseline trials, results revealed no significant main effect for the factor Group ($F(1,121) = 1.211$, $p = .292$, partial $\eta 2 = .009$), Group x Experiment ($F(1,121) = .032$, $p = .859$, partial $\eta 2 < .001$), Emotion ($F(1,121) = .680$, $p = .411$, partial $\eta 2 = .006$) or Emotion x Experiment ($F(1,121) = .194$, $p = .660$, partial $\eta 2 = .002$). Results further did not show any significant interaction for Group x Emotion ($F(1,121) = 1.312$, $p = .254$, partial $\eta 2 = .011$) or Group x Emotion x Experiment ($F(1,121) = 1.082$, $p = .300$, partial $\eta 2 = .009$).
