# Supplementary material for: The Modulation of Mimicry by Ethnic Group-Membership and Emotional Expressions
Source: PLoS One. 2016 Aug 24;11(8):e0161064. doi: 10.1371/journal.pone.0161064 (PMC4996423; doi:10.1371/journal.pone.0161064)
Supplement: S4 File — Mimicry effect, Response Facilitation and Inhibition including the factor Happy (SAMT with vignettes). (PDF) [file pone.0161064.s004.pdf]

S4 File

Additional results experiment 3

Mimicry effect, response facilitation and inhibition including the factor *Happy* (SAMT with vignettes)

Mimicry effect

We performed a with a repeated measurement ANOVA with factors GROUP (In-group, Out-group) and THREAT (Happy, Angry/No Personal Threat, Angry/Personal Threat) on the mean difference scores (i.e., RT on incongruent minus congruent trials). This revealed a trend significant GROUP X THREAT interaction ($F(2,114) = 4.386$, $p = .015$, partial $\eta 2 = .071$) in the absence of significant main effects (all ps $\leq .26$). We carried out planned pairwise comparisons which showed a significantly stronger mimicry effect when Out-group / Personal Threat stimuli were presented than In-group Personal Threat stimuli ($t(57) = 22.76$, $p = .008$, see Table 1 for details). Moreover, the effect of type of threat was different for the In-group, as shown by a significant difference when In-group / No Personal Threat was compared to In-group / Personal Threat, with a higher mimicry effect for In-group / No Personal Threat ($t(57) = 3.34$, $p = .001$, see Table 1 for details). Also the mimicry effect for In-group Happy was higher than for In-group Threat ($t(57) = 2.023$, $p = 0.048$). No other significant differences were found (all ps $\geq .303$).

We performed a three-way repeated measures ANOVA with the factors THREAT (Happy, Angry/No Personal Threat, Angry/ Personal Threat), GROUP (In-, Out-Group) and CONGRUENCY (Congruent, Incongruent). This revealed a main effect for CONGRUENCY ($F(1,57) = 400.522$, $p \leq .001$, partial $\eta 2 = .875$) with faster mean reaction times for congruent compared to incongruent trials (congruent: $M = 476.243$, $SE = 8.04$; incongruent: $M = 546.655$, $SE = 9.702$).

Response facilitation

We performed repeated measures ANOVAs separate on congruent trials with the factors THREAT (Happy, Angry/No Personal Threat, Angry/ Personal Threat), GROUP (In-, Out-Group). This revealed a trend significant GROUP X THREAT interaction ($F(1,114) = 3.008$, $p = .053$, partial $\eta 2 = .05$), in the absence of significant main effects (all ps $\geq .43$). Planned comparisons revealed no further significant differences when adding the presentation of happy faces to analyses (all *p's* $\geq .163$).

Response inhibition

We performed repeated measures ANOVAs separate on incongruent trials with the factors THREAT (Happy, Angry/No Personal Threat, Angry/ Personal Threat), GROUP (In-, Out-Group). This revealed no significant main effects for GROUP ($F(1,57) = .49$, $p = .487$, partial $\eta 2 = .009$) and a trend significant main effect for THREAT ($F(1,114) = 2.823$, $p = .064$, partial $\eta 2 = .047$). No significant effect was found for the interaction GROUP X THREAT ($F(1,114) = .226$, $p = .798$, partial $\eta 2 = .004$).
